# A Machine Learning Approach to Predict Watershed Health Indices for Sediments and Nutrients at Ungauged Basins

**DOI:** 10.3390/w15030586

**Published:** 2023

**Authors:** Ganeshchandra Mallya, Mohamed M. Hantush, Rao S. Govindaraju

**Affiliations:** 1 Lyles School of Civil Engineering, Purdue University, West Lafayette, IN 47907, USA; 2 U.S. EPA Center for Environmental Solutions and Emergency Response, 26 West Martin Luther King Dr., Cincinnati, OH 45268, USA

**Keywords:** machine learning, water quality, risk analysis, ungauged watersheds, random forest regression, AdaBoost, gradient boosting, Bayesian ridge regression

## Abstract

Effective water quality management and reliable environmental modeling depend on the availability, size, and quality of water quality (WQ) data. Observed stream water quality data are usuallEEy sparse in both time and space. Reconstruction of water quality time series using surrogate variables such as streamflow have been used to evaluate risk metrics such as reliability, resilience, vulnerability, and watershed health (WH) but only at gauged locations. Estimating these indices for ungauged watersheds has not been attempted because of the high-dimensional nature of the potential predictor space. In this study, machine learning (ML) models, namely random forest regression, AdaBoost, gradient boosting machines, and Bayesian ridge regression (along with an ensemble model), were evaluated to predict watershed health and other risk metrics at ungauged hydrologic unit code 10 (HUC-10) basins using watershed attributes, long-term climate data, soil data, land use and land cover data, fertilizer sales data, and geographic information as predictor variables. These ML models were tested over the Upper Mississippi River Basin, the Ohio River Basin, and the Maumee River Basin for water quality constituents such as suspended sediment concentration, nitrogen, and phosphorus. Random forest, AdaBoost, and gradient boosting regressors typically showed a coefficient of determination $R^{2}>0.8$ for suspended sediment concentration and nitrogen during the testing stage, while the ensemble model exhibited $R^{2}>0.95$. Watershed health values with respect to suspended sediments and nitrogen predicted by all ML models including the ensemble model were lower for areas with larger agricultural land use, moderate for areas with predominant urban land use, and higher for forested areas; the trained ML models adequately predicted WH in ungauged basins. However, low WH values (with respect to phosphorus) were predicted at some basins in the Upper Mississippi River Basin that had dominant forest land use. Results suggest that the proposed ML models provide robust estimates at ungauged locations when sufficient training data are available for a WQ constituent. ML models may be used as quick screening tools by decision makers and water quality monitoring agencies for identifying critical source areas or hotspots with respect to different water quality constituents, even for ungauged watersheds.

## 1. Introduction

The quality of water resources, be it streams, rivers, lakes, reservoirs, ground water, or oceans, determines the well-being of human populations and other natural or physical systems that rely on it. Extensive changes to land use, e.g., from forest or grass land to agricultural or urban land use, may result in detrimental effects on both the quantity and the quality of water [1]. For example, agriculture activities such as tilling, application of fertilizers and pesticides, and lack of cover during non-growing seasons can all lead to the pollution of local streams and rivers, as well as final receiving waters such as lakes, seas, and oceans [2,3]. Urban activities such as lawn maintenance and landscaping, transportation, construction, and untreated domestic and industrial wastewater also lead to the pollution of water resources [4]. Fecal indicator bacteria such as Escherichia coli can be part of runoff pollution and can contaminate waters in the nearshore and coastal regions [5]. When the levels of nitrate + nitrite are above permissible limits in the streams, they can pose both non-carcinogenic and carcinogenic risks to public health and can affect general ecosystem functioning [6]. When water is polluted, it often results in health issues among human beings, animals, plants, etc., as well as affecting several ecosystem services [7].

As sampling and laboratory analyses can be expensive and time consuming, it is often impossible to get sufficient spatial and temporal coverage of water quality (WQ) samples for assessment. Therefore, water resources professionals have to rely on techniques such as time series reconstruction using statistical [8,9] or physically based models [10,11] to overcome the limitation of sparse water quality data. However, popular distributed water quality models such as HSPF (hydrologic simulation program, Fortran; [11]) and SWAT (soil and water assessment tool; [10]), are often challenged by the lack of sufficient data for calibration, computer effort, and user expertise. These models do not lend themselves for use in ungauged basins. Deploying any of the above models as a quick and simple screening tool to identify potential source areas for contaminants might be overkill.

Several studies have used machine learning (ML) models for identifying water bodies from satellite images [12], water demand forecasting [13], sediment transport modeling [14–16], ecological modeling [17–21], estimating design floods [22], flood susceptibility and risk assessment [23,24], flood prediction [25,26], outbreak of harmful algal blooms [27], ML-based satellite image analysis [28,29], as well as water quality assessment [30–44].

Risk-based frameworks have been used in several studies to evaluate the health of water resources systems such as reservoirs [45] and watersheds [8,46,47]. More recently, Mallya et al. [48] proposed a new vulnerability risk measure and composite watershed health index that conveniently ranges between zero and one and uses reliability, resilience, and vulnerability (R-R-V) risk metrics in its computation. A value of zero indicates poor watershed health with respect to a chosen water quality parameter, whereas a value of one indicates high watershed health. However, decisions are desired at different scales and for ungauged watersheds. To address this need, the following are the study objectives:

1. To evaluate machine learning (ML) models to predict watershed health at ungauged basins with respect to suspended sediment concentration (SSC) and nutrients (nitrogen and phosphorus). ML models, namely random forest, AdaBoost, gradient boosting regressor, and Bayesian ridge regression, were chosen in this study because these models do not make any assumptions about input data distributions, they work well with high dimensional datasets, and they avoid overfitting by using random combinations of predictor variables to develop uncorrelated set of models.
2. To identify predictors such as watershed attributes (e.g., drainage area, stream order, drainage density, watershed slope, etc.), long-term climate data (monthly, seasonal and annual precipitation, and/or temperature data), soil data, land use/land cover data, and fertilizer sales data to train machine learning models for predicting watershed health over any area of interest within three Midwest river basins.
3. To develop spatial maps of watershed health at HUC-10 resolution to aid decision makers in identifying critical source areas in the Midwest river basins.

## 2. Study Area and Datasets

**Figure 1.**
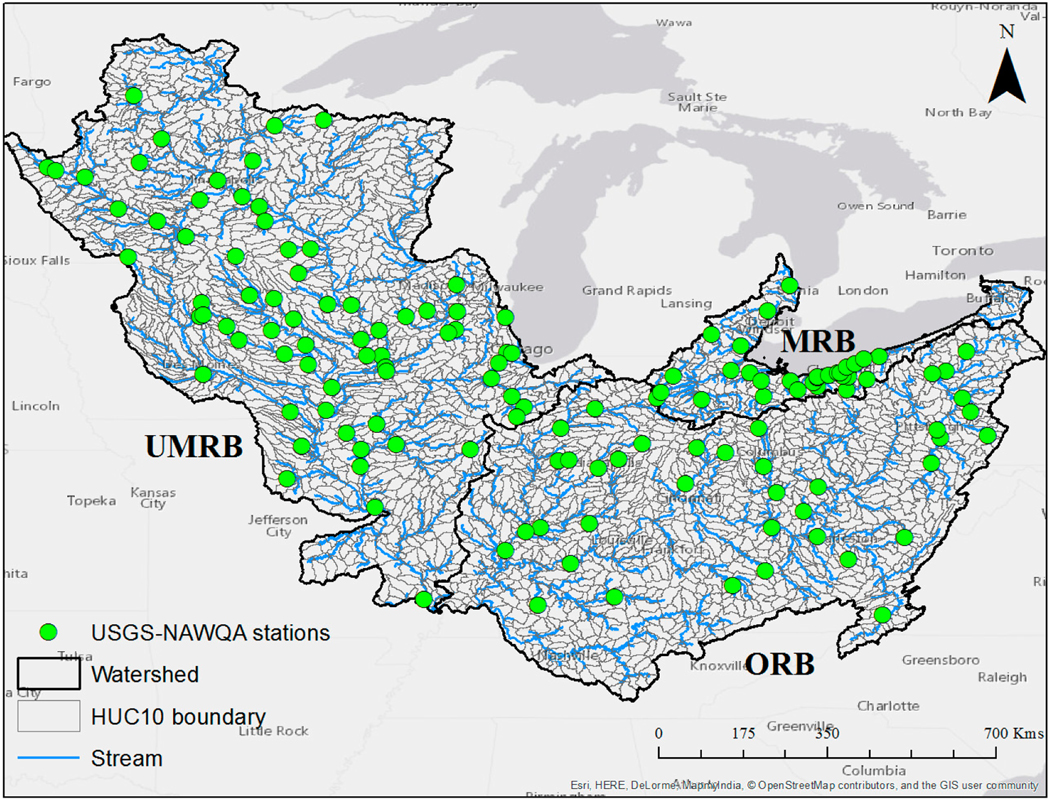
Study area consists of the Upper Mississippi River Basin, the Ohio River Basin, and the Maumee River Basin. Green circular markers denote the geographic location of USGS-NAWQA stations, where data for water quality constituents such as suspended sediment concentration, nitrogen, and phosphorus are available.

The study area (see Figure 1) consists of the Upper Mississippi River Basin (UMRB), the Ohio River Basin (ORB), and the Maumee River Basin (MRB or the Lake Erie watershed, USA side only). These river basins are spread across multiple states located in Midwest and Northeastern United States. The drainage areas for UMRB, ORB, and MRB are 490,000 square kilometers, 490,600 square kilometers, and 56,926 square kilometers, respectively. These river basins have dominant agricultural land use, therefore there is keen interest in studying the quantity and quality of water resources over the region.

The following datasets were used in this study: (i) streamflow from the United States Geological Survey (USGS) daily streamflow dataset and water quality data from the United States Geological Survey-National Water Quality Assessment (USGS-NAWQA) stations, (ii) geographic data such as Hydrologic Unit Code 10 (HUC-10) boundaries and stream network shapefiles from the USGS National Hydrography Dataset (NHD), (iii) land use data from the 2011 National Land Cover Database (NLCD), (iv) soil data such as hydrologic soil group and available water storage (AWS) in the top 25 cm of soil from the Soil Survey Geographic Database (SSURGO), (v) precipitation (PRCP) and minimum and maximum temperature (TMIN and TMAX) data from the Historical Climate Network (HCN) stations, (vi) fertilizer sales data available from the United States Department of Agriculture’s National Agricultural Statistics Service (USDA-NASS), and (vii) drainage areas, slope, stream order, and latitude and longitude information from 30 m resolution digital elevation model data (DEM). Readers are referred to the Supplementary Information for additional details on the datasets used in the study.

## 3. Methodology

### 3.1. Risk and Watershed Health Measures

The mathematical formulation of risk and the composite watershed health measures used in this study are described here.

Reliability (p) is defined as the probability of the system to be in a compliant state (S):

(1) $$p=1-P\left\{X_{t}\in F\right\}=1-\frac{1}{n}\sum_{t=1}^{n}z_{t}$$

where $X_{t}$ is the water quality concentration at time t, $z_{t}=1$ when $X_{t}\in F$ and 0 when $X_{t}\in S$, and n is the total number of data points. The state is said to be non-compliant (F) when the water quality exceeds the user defined standard concentration $\left(X^{*}\right)$, i.e., $X_{t}>X^{*}$.

Resilience (r) is defined as the probability of the system to recover from a non-compliant state:

(2) $$r=P\left\{X_{t+1}\in S|X_{t}\in F\right\}=\frac{P\left\{X_{t+1}\in S\cap X_{t}\in F\right\}}{P\left\{X_{t}\in F\right\}}=\frac{\sum_{t=1}^{n}y_{t}}{\sum_{t=1}^{n}z_{t}}=\frac{l}{m}$$

where $y_{t}=1$ when $X_{t+1}\in S$ and $X_{t}\in F$ and 0 otherwise, $z_{t}=1$ when $X_{t}\in F$ and 0 when $X_{t}\in S$, *m* is the number of instances the water quality standard is violated $\left(m=\sum_{t=1}^{n}z_{t}\right)$, and l is the number of transitions from non-compliant to compliant state or $l=\sum_{t=1}^{n}y_{t}$.

Vulnerability is the magnitude of damage during a non-compliant event. During risk analysis for a water quality constituent, vulnerability may denote the total magnitude of violations or damage caused in dollar amounts. In order for the vulnerability and thus the watershed health measures to range from 0 to 1 for consistency, Mallya et al. [48] introduced a new measure called *opposite of vulnerability*:

(3) $$v=\exp\left\{-\frac{1}{m}\sum_{t=1}^{n}\log\left[\frac{Q_{t}X_{t}t}{Q_{t}X^{*}t}\right]H\left[X_{t}-X^{*}\right]\right\}$$

The Heaviside step function H[.] is equal to 1 when $X_{t}>X^{*}$ and 0 otherwise. The terms $Q_{t}X_{t}t$ and $Q_{t}X^{*}t$ represent water quality load and the standard load at time t, respectively. When the deviations of $X_{t}$ from $X^{*}$ are large, v is closer to zero and, when the deviations are small, v is closer to 1. Note that Equation (3) can be written as

(4) $$v=\prod_{i=1}^{m}{\left(\frac{Q_{i}X^{*}\text{Δ}t}{Q_{i}X_{i}\text{Δ}t}\right)}^{\frac{1}{m}}$$

where i refers to the ith time instance where $X_{t}>X^{*}$ and m is the number of violation instances. Note that when an upper threshold value is not to be exceeded as the water quality criterion, e.g., for oxygen, Equation (4) becomes $v=\prod_{i=1}^{m}{\left(\frac{Q_{i}X_{i}\text{Δ}t}{Q_{i}X^{*}\text{Δ}t}\right)}^{\frac{1}{m}}$, where i refers to the ith time instance where $X_{t}<X^{*}$. Equation (4) implies that v is the geometric mean of the severity of the violations.

Vulnerability can be calculated as:

(5) Vul=1−v

Using the risk indices described above, Mallya et al. [48] proposed a conservative measure for watershed health:

(6) $$h={\left(p\times r\times v\right)}^{\frac{1}{3}}$$

When p=r=v=1, h=1 (i.e., watershed is healthy) and, when one of them is zero, h=0 (i.e., watershed is unhealthy).

### 3.2. Machine Learning Models

In this study, four machine learning models, namely, random forest, AdaBoost, gradient boosting, and Bayesian ridge regression were adopted to predict watershed health indices for sediments and nutrients at ungauged basins. Brief mathematical descriptions of these models are presented below.

#### 3.2.1. Decision Trees

Decision trees [49] are a non-parametric supervised learning technique used for classification and regression problems. Decision trees can handle mixed data types (numerical and categorical) and can model complex functions. The goal is to develop a model that predicts the value of a response variable by learning simple decision rules inferred from the predictor vectors. Given predictor vectors $x_{j}\in R^{n}$, j=1,…,p and response vector y∈R, a decision tree recursively partitions the space to obtain groupings of samples that meet certain threshold criteria $\left(t_{m}\right)$ at each node of the decision tree.

Let the data at node m be represented by D. Then, at this node for each candidate split $\theta =\left(j,t_{m}\right)$ consisting of feature j and threshold $t_{m}$, partition the data into $D_{left}(\theta )$ and $D_{right}(\theta )$ such that:

(7) $$D_{left}(\theta )=(x,y)|x_{j}\leq t_{m}$$

(8) $$D_{left}(\theta )=D\setminus D_{\text{left}}(\theta )$$

Thus, $D_{right}(\theta )$ contains all the elements of the set D that are not in $D_{left}(\theta )$. The impurity at m is computed as:

(9) $$G(D,\theta )=\frac{n_{left}}{N_{m}}H\left(D_{left}(\theta )\right)+\frac{n_{right}}{N_{m}}H\left(D_{right}(\theta )\right)$$

where $n_{left}$ and $n_{right}$ denote the number of samples in each partition [$D_{right}(\theta )$ and $D_{left}(\theta )$], $N_{m}$ is the number of samples at node m and the function H() is the mean squared error in regression problems, i.e., if

(10) $$c_{m}=\frac{1}{N_{m}}\sum_{i\in N_{m}}y_{i}$$

(11) $$H\left(X_{m}\right)=\frac{1}{N_{m}}\sum_{i\in N_{m}}{\left(y_{i}-c_{m}\right)}^{2}$$

(12) $$\theta^{*}=argmin_{\theta }G(D,\theta )$$

One selects the parameters that minimize the impurity where *argmin* refers to the argument of the minimum, i.e., set of values $\theta^{*}$ for which the function G() attains the smallest value. Recursion for subsets $D_{left}\left(\theta^{*}\right)$ and $D_{right}\left(\theta^{*}\right)$ follows until the maximum allowable depth for the tree is reached and $N_{m}<min_{samples}$ or $N_{m}=1$.

#### 3.2.2. Random Forest

Random forest (RF; [50]) consists of an ensemble of decision trees $\left\{T_{1}(X),\ldots ,T_{B}(X)\right\}$, where $X=\left\{x_{1},\ldots ,x_{p}\right\}$ is a p-dimensional vector of attributes for an annual data point that belongs to a drainage area or a HUC-10 basin. The RF model generates B outputs $\left\{{\hat{y}}_{1}=T_{1}(X),\ldots ,{\hat{y}}_{B}=T_{B}(X)\right\}$, where ${\hat{y}}_{b},b=1,\ldots ,B$, is the prediction for an annual sample by the $b^{th}$ tree. The average output of all B trees are reported as the final prediction, $\hat{y}$.

Suppose we have a total of N annual samples (a collection of annual data from all stations in the study area where the chosen water quality constituent is monitored) in the training set, then we can represent the explanatory variables ($X_{i}$, i=1,…,N), where $X_{i}=\left(x_{1i},x_{2i},\ldots ,x_{pi}\right)$ and target variable $\left(y_{i}\right)$ as $D=\left\{\left(X_{1},y_{1}\right),\ldots ,\left(X_{N},y_{N}\right)\right\}$. The random forest model works as follows:

1. First N random samples (X, y) are drawn with replacement (also known as bootstrap-ping or bagging). Here, the assumption is that the N samples are independent and identically distributed.
2. Using these N samples, we train a decision tree such that, at each node, the best split is decided using a randomly selected subset of $m_{try}$ attributes. An unpruned decision tree may be grown such that no further splits are possible at the end nodes. However, in this approach we treated both the depth of the tree and the number of attributes ($m_{try}$) used at each node as variables.
3. The above two steps are repeated until B decision trees are grown.

The RF algorithm is quite robust, even when we have several hundreds, if not thousands, of explanatory variables. As individual trees are grown using a small random subset of attributes ($m_{try}$), the trees within the forest are not only relatively unique but the time required to train a RF model is also reduced compared with that of a single decision tree (DT). The variable $m_{try}$ was treated as a model parameter in this study and an optimum value was found using a 5-fold cross validated grid search. If one chooses to avoid setting this as a model parameter, then the recommended value for $m_{try}$ is the square root of the number of attributes (i.e., $\sqrt{p}$). Typically, the RF model does not have to be pruned, i.e., it can be grown to maximum depth without loss of generalization or overfitting [49] but, to obtain fairly unique trees (that result in a robust RF model), the depth of the tree was also treated as a model parameter and was tuned using the grid search approach.

In this study, for consistency the performances of all machine learning models were evaluated on an independent test data set that was not used during training. However, in many applications (including this study) the data available are limited. To address this limitation, a k-fold cross-validation (CV) approach (where the model is trained on k-1 subsets, tested on the kth subset, and repeated k times or until each subset is used for testing) was used. The RF model internally performs cross-validation during the training phase using out-of-bag (OOB) samples. Specifically, as bootstrapped samples are used for training each decision tree, the unused samples, referred to as OOB samples, are used to evaluate the performance of each tree within the RF model. The mean square error (MSE) (or other similar error metrics) on OOB predictions is calculated for each DT and then the average mean error statistic from the k-fold CV is reported for the model. A trained DT can provide easy-to-comprehend relationships between the attributes and the target and therefore reveals both important attributes and those that have least predictive power. Since a RF model is an ensemble of DTs, it can also reveal the importance of the attributes.

#### 3.2.3. AdaBoost

The boosting algorithm AdaBoost [51] is used to fit a sequence of weak learners, such as small decision trees, on continuously modified versions (by weighting) of the training data. The predictions from each learner are then combined through a weighted summation (or voting in case of classification problems) to produce the final prediction. The data are modified after each boosting process by applying weights $w_{1},w_{2},\ldots ,w_{N}$ to each training sample, where N is the total number of samples. During the first iteration, the weights, $w_{i\ldots N}$, are all set to $\frac{1}{N}$. For each successive iteration, the sample weights are modified [49] and the learning algorithm is reapplied to the re-weighted data. At the end of any boosting step, training examples that were incorrectly predicted by the boosted model will have their weights increased, whereas the weights are decreased for those examples that were predicted correctly. Therefore, for every iteration, the weak learner is forced to concentrate on the examples that were missed previously. For regression problems, a weak regressor such as a decision tree regressor [49] is used to obtain a fit on the dataset. Then, additional copies of the regressor are fit on the same dataset but where the weights of instances are adjusted according to the error of the current prediction. As such, subsequent regressors focus more on difficult cases.

#### 3.2.4. Gradient Boosting

Gradient boosting regression trees [49] use an additive model of the form:

(13) $$F(X)=\sum_{m=1}^{M}h_{m}(X)$$

where $h_{m}(X)$ are weak learners, such as decision trees, F(X) is the final model, and M is the total number of weak learners. Similar to other boosting algorithms, gradient boosting involves using a forward stage wise additive model:

(14) $$F_{m}(X)=F_{m-1}(X)+h_{m}(X)$$

For every iteration, a weak learner (such as the decision tree) $h_{m}(X)$ is chosen to minimize the loss function $L_{m}$ (least squares) given the current model $F_{m-1}$.


$$h_{m}=\underset{h}{\text{argmin}}L_{m}=\underset{h}{\text{argmin}}\sum_{i=1}^{n}l\left(y_{i},F_{m-1}\left(x_{i}\right)+h_{m}\left(x_{i}\right)\right)$$

Using first-order Taylor approximation

$$l\left(y_{i},F_{m-1}\left(x_{i}\right)+h_{m}\left(x_{i}\right)\right)\approx l\left(y_{i},F_{m-1}\left(x_{i}\right)\right)+h_{m}\left(x_{i}\right){\left[\frac{\partial l\left(y_{i},F\left(x_{i}\right)\right)}{\partial F\left(x_{i}\right)}\right]}_{F=F_{m-1}}$$

The term ${\left[\frac{\partial l\left(y_{i},F\left(x_{i}\right)\right)}{\partial F\left(x_{i}\right)}\right]}_{F=F_{m-1}}$ may be denoted as $g_{i}$, which is the derivative of the loss with respect to the second parameter evaluated at $F_{m-1}(x)$. Removing the constant terms results in:

$$h_{m}\approx \underset{h}{\text{argmin}}\sum_{i=1}^{n}h\left(x_{i}\right)g_{i}$$

This minimization problem is solved numerically using steepest gradient descent [52].

#### 3.2.5. Bayesian Ridge Regression

A Bayesian ridge regression is a probabilistic model of the regression problem of the form [53]:

(15) p(y∣X,w,α)=N(y∣Xw,α)

The prior for the parameter *w* may be chosen as a spherical Gaussian with precision $\lambda^{-1}$.


$$p(w|\lambda )=N\left(w|0,\lambda^{-1}I_{p}\right)$$

By choosing this prior, the Bayesian ridge regression uses regularization with L2-norm, which means that the outliers are assigned more weights. Alpha (α) is a regularization parameter that controls the amount of shrinkage. It is treated as a random variable and is estimated from the data. Large values of alpha result in greater shrinkage, thus making the coefficients (w) sparser and more robust to collinearity. The priors over α and λ are chosen to be gamma distributions. The parameters of the model are estimated by maximizing the marginal log likelihood [54].

The machine learning models described in this section are available in the Scikit-learn machine learning toolbox for Python [55]. For models that use decision trees as weak learners, i.e., random forests, AdaBoost, and gradient boosting regressor, the value of the *max_features* parameter (where *max_features* are the number of predictor variables to consider when looking for the best split of each decision tree) was found to be optimum at p/3, using the grid search approach [56]. Here, p refers to the total number of attributes or explanatory variables in the input dataset.

## 4. Results and Discussion

### 4.1. Prediction of Watershed Health

Following Hoque et al. [8], continuous daily time series of streamflow data and sparse observations of water quality data recorded at USGS-NAWQA stations were used to obtain a reconstructed continuous time series of daily water quality load using relevance vector machines. Then, using user-defined standards for each water quality parameter, the above-described risk and watershed health measures were computed for each year during the study period (1966–2014). The computed watershed health index (WH) for each water quality parameter formed the target vector y during the training phase of the machine learning models. Watershed attributes, long-term climate data, and soil and land-use data compiled over each area draining to USGS-NAWQA stations formed the predictor series X. During the training phase, data (X and y) from 80% of the stations were used. Each model performance was validated using data from the remaining 20% of the stations to obtain the annual time series of the watershed health predictions $\left(y_{pred}\right)$. During the training phase (i.e., using data from 80% of the stations), the robustness of the trained machine learning models was validated using five-fold cross validation (CV), see Table 1. Using the ensemble model, the WH index with respect to each constituent (sediment and nutrients) was predicted for ungauged HUC-10 basins using predictor variables (X) compiled over these ungauged basins.

#### 4.1.1. Watershed Health for Suspended Sediment Concentration

SSC load was first reconstructed following Hoque et al. [8] to obtain daily continuous time series of SSC loads at each of the 151 USGS-NAWQA stations in the study area where sparse SSC observations were available. Then, by setting the WQ standard to 30 mg/L [57], risk measures such as R-R-V and WH measures were computed for each year during the study period. This annual series of watershed health (WH) index (with respect to SSC) at each USGS-NAWQA station forms the target series (y) for training machine learning models. The data for predictor variables (X) (except fertilizer sales, as SSC is not directly influenced by fertilizer application) that were compiled for each drainage area of USGS-NAWQA station were used during the training and testing phase; those that were compiled for ungauged HUC-10 basins were used during the prediction phase.

##### Random Forest Regression Model

A random forest regression model with 500 decision trees was used for predicting watershed health at ungauged HUC-10 basins. The model was first trained using data available at USGS-NAWQA stations. As described above, the annual series of WH measure $\left(y=\left(y_{1},\ldots ,y_{N}\right)\right)$ and 81 explanatory variables (X, including watershed attributes, climate, soil, and land-use data) at about 80% of the USGS-NAWQA stations (out of total 151 stations) were used for training. A five-fold CV was performed to check the robustness of the trained model. The model performance was evaluated using the coefficient of determination, $R^{2}$, which was found to have an average value of 0.98 from the five-fold CV (Table 1).

The trained model was then confirmed using the test dataset. An R^2^ for the test set is shown between parentheses in Table 1. Watershed attributes (X) at the remaining 20% of the stations were used as inputs to the trained model and the predicted WH values were compared with those values computed from observed WQ data obtained from gauging/sampling stations following Mallya et al. [48]. Henceforth, we refer to WH values computed from observed WQ data as reference WH values. An $R^{2}$ value of 0.95 was obtained on the test set. Though the $R^{2}$ statistic is high, there was significant scatter about the best-fit (1:1) line indicating under- or overprediction for some cases. Similar RF model test performance for risk measures reliability, resilience, and vulnerability of SSC was obtained.

**Figure 2.**
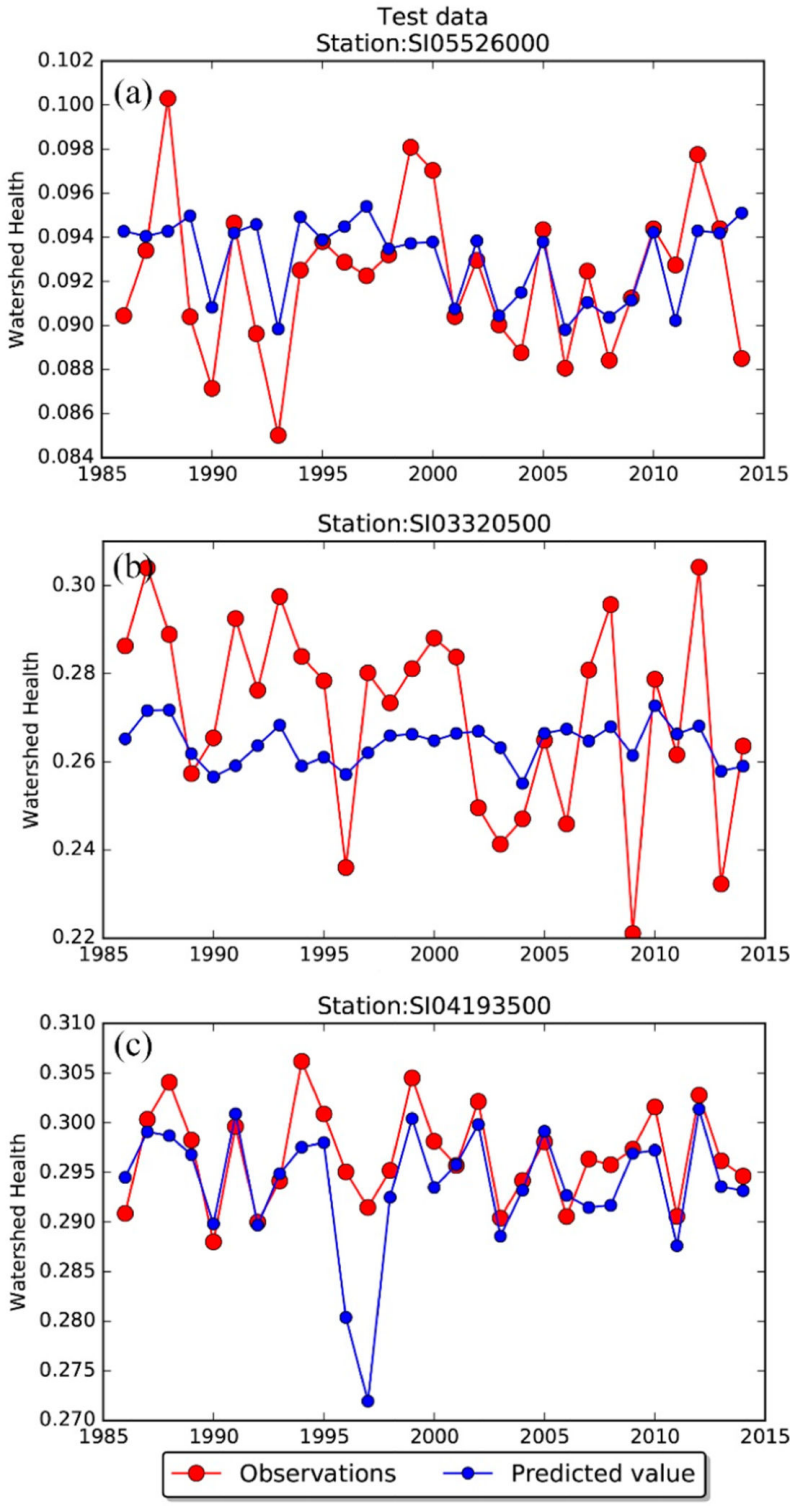
Time series comparison of reference watershed health values inferred from measurements as in Mallya et al. [48] versus predicted watershed health (with respect to SSC) using a random forest model at USGS-NAWQA stations (**a**) 05526000 Iroquois River near Chebanese, IL, (**b**) 03320500 Pond River near Apex, KY, and (**c**) 04193500 Maumee River at Waterville, OH that were used in the test set. Only values for the period 1986–2014 are shown for brevity.

As an example, over three USGS-NAWQA stations (each belonging to one of the three river basins UMRB, ORB, and MRB), the time series comparison of reference WH versus predicted watershed health measures (with respect to standard chosen for SSC, i.e., 30 mg/L) is presented in Figure 2a–c. The reference WH values are denoted using a line plot with red markers, and the predictions from the random forest model are shown using a line plot with blue circular markers. While some predictions are close to the reference values (notice that the y-axis is zoomed in to highlight the differences; if the y-axis is scaled between zero and one then these differences do not get highlighted), they are either overpredicted in some instances (Figure 2a) or underpredicted (Figure 2b) during other instances. In addition, the annual pattern (rise and fall) of watershed health variation during the study period may not necessarily be captured (for example, see Figure 2a,b vs. Figure 2c).

**Figure 3.**
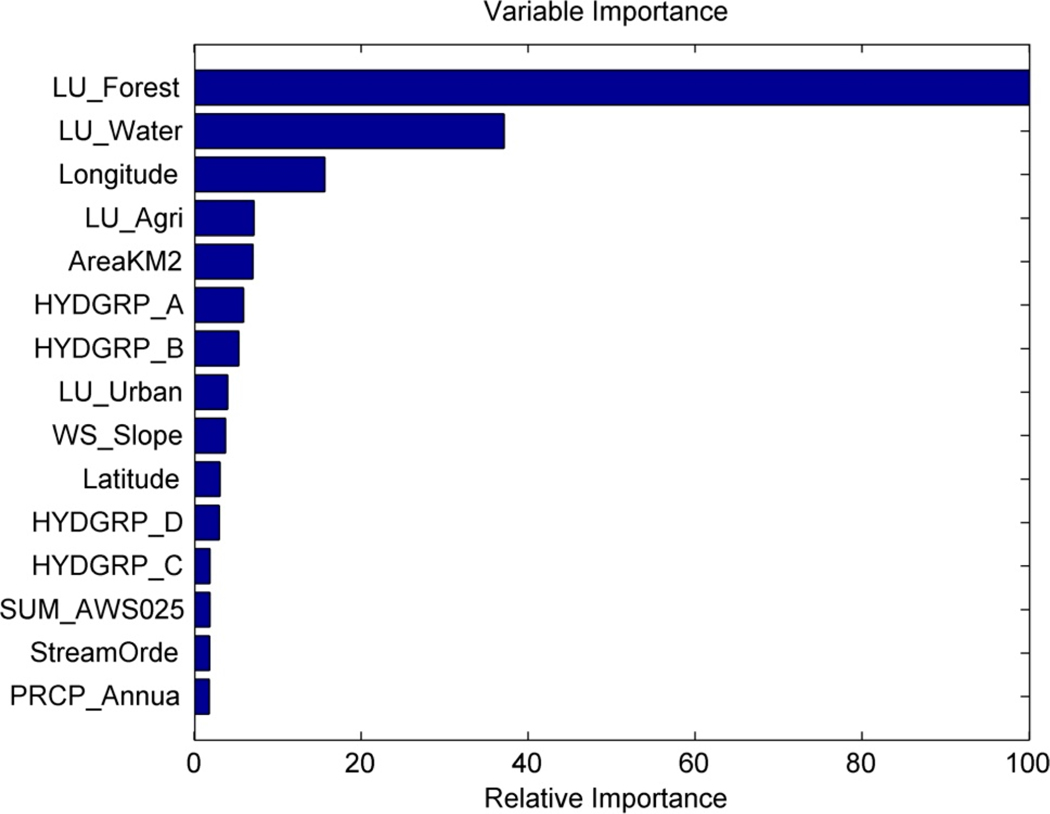
Variable importance for top 15 out of 81 explanatory variables according to random forest model trained on watershed health (with respect to SSC) at USGS-NAWQA stations.

The random forest regression model also computes the impurity or error (e.g., root mean squared error) at different nodes of all decision trees in the model during the training phase (five-fold CV) by removing one explanatory variable at a time. The overall errors are compiled for each variable and then sorted and scaled between 0 and 100%. The variable that results in the highest model error is the most important and vice-versa. Figure 3 shows the top 15 (out of 81) variables that were important in explaining watershed health (with respect to SSC) according to the random forest model. The percentage areas under forest, water, and agricultural land use were among the top five variables, along with drainage area and geographic location. Among the climate variables, annual precipitation and annual average values of maximum and minimum temperature were most influential. Percentages of forest land use (less susceptible to soil erosion), agricultural land use (source of sediments, although depending on soil conservation practices), and longitude were identified as important predictors for watershed health (with respect to SSC) over the three river basins (Table 1).

**Figure 4.**
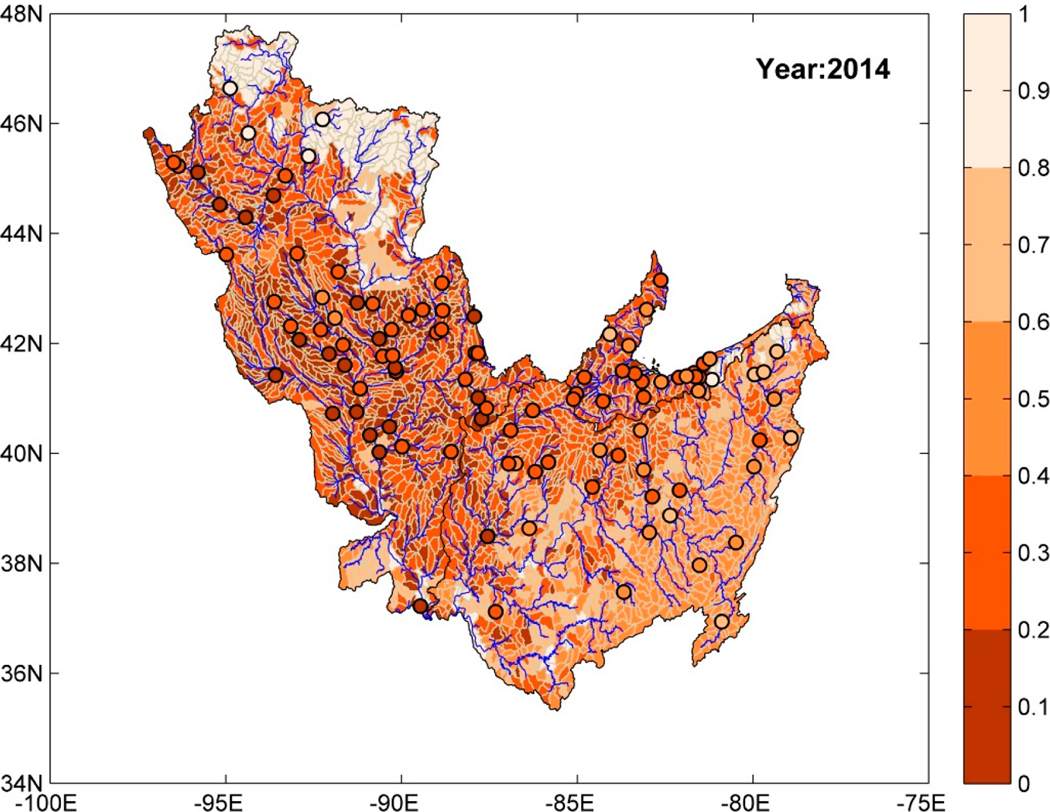
Prediction of watershed health (with respect to SSC) at ungauged HUC-10 basins for the year 2014 using random forest regression model. Circular markers denote the location of USGS-NAWQA stations where SSC measurements were available and are color-coded based on watershed health for year 2014.

After the training and testing phases, the random forest model was used to obtain predictions of risk measures (annual series) at HUC-10 basins using attributes (X) collected over these ungauged basins as an input to the model. Figure 4 shows a map of the study region, where each HUC-10 basin is color-coded using predictions of watershed health (with respect to SSC) for the year 2014. The circular markers denote the location of stations where SSC observations were available. The circular makers are also color-coded with watershed health values for 2014 computed using daily reconstructed SSC series for that year. Five different colors for watershed health increments of 0.2 were used in Figure 4. The lightest shade represents high watershed health and the darkest shade represents low watershed health. The HUC-10 basins located in the uppermost reaches of the river basins have high watershed health that deteriorates for downstream watersheds. In addition, the HUC-10 basins with high watershed health coincide with areas dominated by forest land use, e.g., north of UMRB, east of MRB, and east and southeast of ORB. Likewise, those with low watershed health belonged to regions with dominant agricultural land use.

##### Other Regression Models

The performance of gradient boosting, adaptive boosting (AdaBoost), and Bayesian ridge regression models for predicting risk measures at ungauged HUC-10 basins is shown in Table 1 and is also discussed in the Supplementary Information. During the testing phase, the gradient boosting regression model yields a $R^{2}$ value of 0.94, which is comparable to that obtained from the random forest model. While most points lie along the best fit line, there is considerable scatter, indicating a lack of strong prediction power. Similarly, the AdaBoost regression model for WH (with respect to SSC) provided a relatively weaker fit with an $R^{2}$ value of 0.84. The Bayesian ridge regression had the worst performance among the four models used in the study, with an $R^{2}$ value of 0.68.

##### Ensemble of Model Outputs

The WH outputs (from the training phase, i.e., 80% of USGS-NAWQA stations) from the four ML models were used as explanatory variables (X) in a separate random forest model with 50 decision trees. The target variable (y) for this model was the annual series of computed WH index, as before. The training and testing were performed in a similar manner using 80–20% split of stations. A five-fold CV was performed during the training phase to evaluate the robustness of the model and an average $R^{2}$ value of 0.98 was achieved. The trained ensemble (random forest) model was then evaluated on the test set. The $R^{2}$ value was 0.98 on the test dataset.

**Figure 5.**
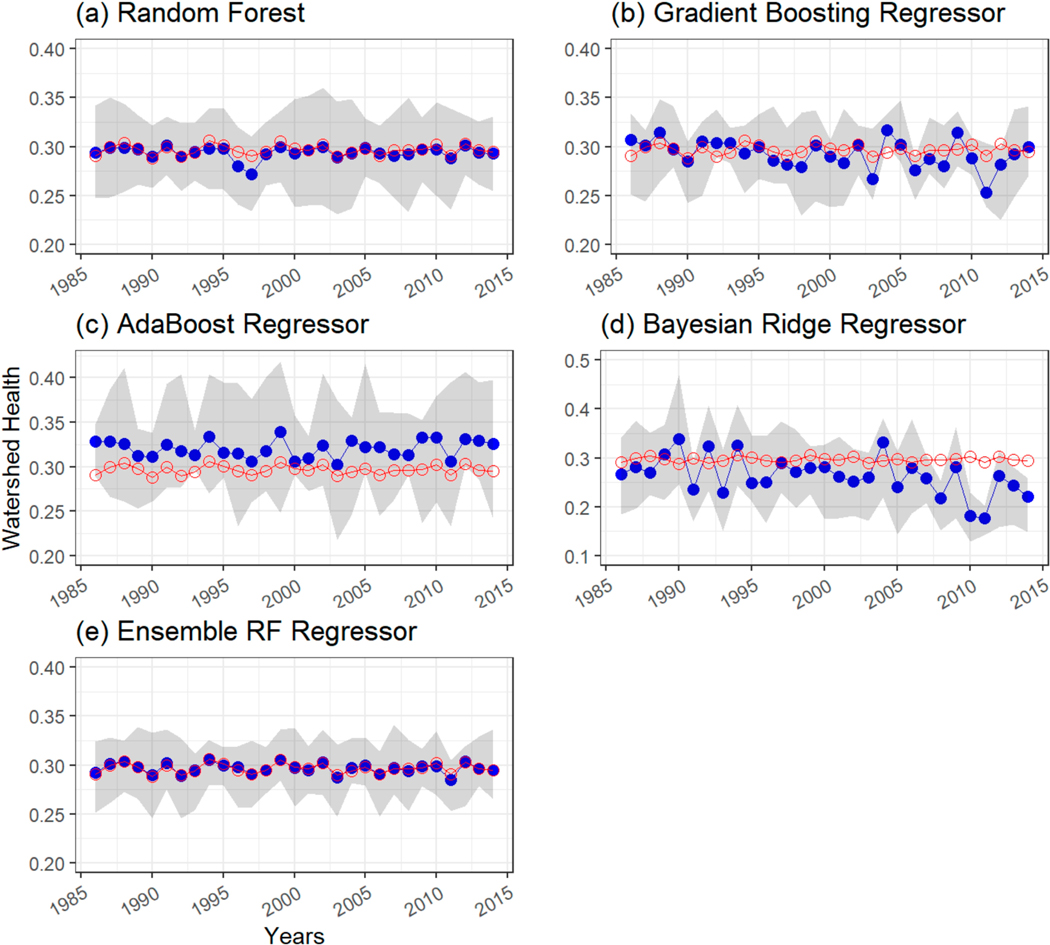
Time series comparison of watershed health (with respect to SSC) estimated from observations as in Mallya et al. [48] at USGS-NAWQA station 04193500 Maumee River at Waterville, OH, which was part of the test set versus ML model predictions using (**a**) random forest regressor, (**b**) gradient boosting regressor, (**c**) AdaBoost regressor, (**d**) Bayesian ridge regressor, and (**e**) ensemble RF regressor. Only values for the period 1986–2014 are shown for brevity. Red-line plot with red-colored hollow circular markers indicates reference WH values. Blue-line plot with solid blue-color circular markers indicates predictions from ML models. The shaded-grey region represents 90 percent prediction interval for the ML models.

The performance of the ensemble model was compared at each station. As an example, Figure 5 shows a comparison of time series plots of reference WH (with respect to SSC) (i.e., computed according to Mallya et al. [48]) and ML model predictions at USGS-NAWQA station 04193500 Maumee River at Waterville, OH, which was part of the test set. The method of Mallya et al. [48] utilizes relevance vector machine to reconstruct SSC (or any WQ constituent of interest) from sparse observations and provides corresponding predictive uncertainty. A Monte Carlo approach provides estimates of WH, which are thus are based on actual observations. The WH series obtained by Mallya et al. [48] serves as a reference for evaluating ML models in this study. Only results for the period 1986–2014 are shown for brevity. In addition, a 90 percent prediction interval for each ML model is shown as the grey-shaded region in the plot. Figure 5a,b indicate that random forest regression and gradient boost regression models predict annual WH values close to the reference WH values derived from observations, as in Mallya et al. [48]. The majority of the reference WH values, shown as red-hollow circular markers, lie within the 90 percent prediction interval bands of these two ML models. Figure 5c shows that the AdaBoost regressor overpredicts WH values (shown as solid-blue circular markers) for most of the years. Figure 5d shows that Bayesian ridge regression performs relatively poorly in predicting annual WH values at this station. The majority of the WH values are underpredicted and some reference points lie outside the 90 percent prediction interval. The ensemble model predictions (Figure 5e) better match the reference WH values compared with individual model predictions (Figure 5a–d). Similar results were observed at other testing stations but have not been included here for brevity.

The trained ensemble model is used for estimating the WH index over ungauged HUC-10 basins using predictions from individual ML models as inputs. The spatial map of ensemble-predicted watershed health is obtained for different years of the study period (1966–2014). In general, the predictions from the ensemble model are similar to those predicted using the random forest model (Figure 4); the HUC-10 basins with high watershed health belong mostly to the regions with dominant forest land use and those with low watershed health are in regions with dominant agricultural land use.

**Figure 6.**
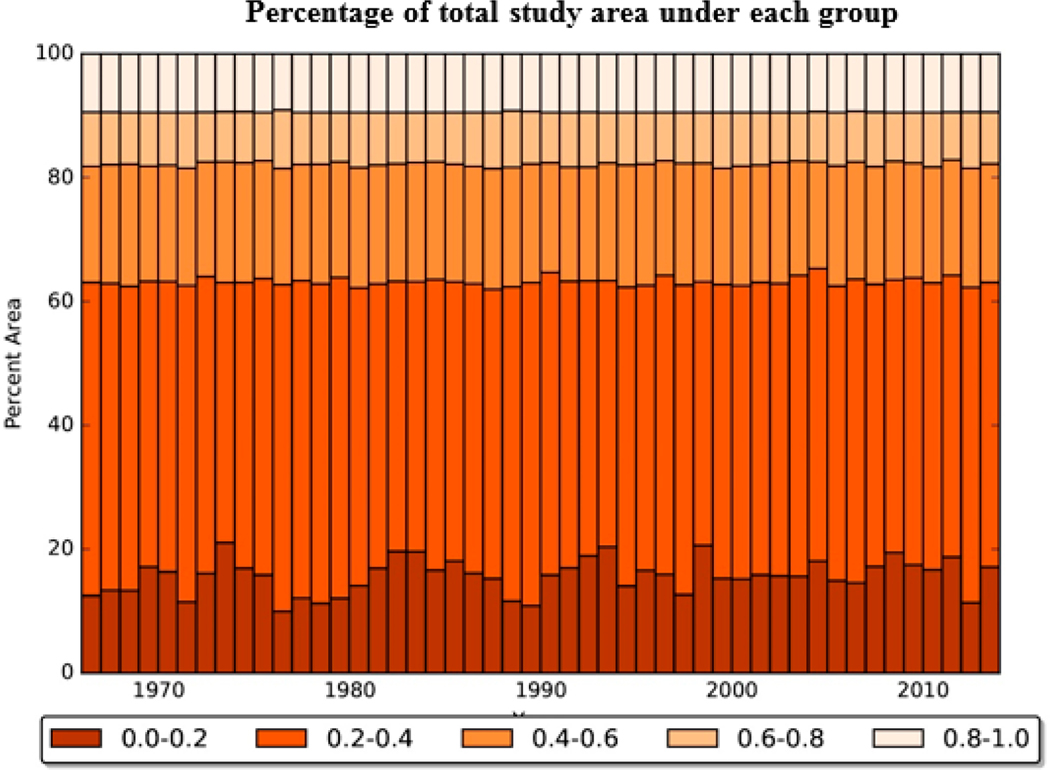
Percentage of total study area under different categories of watershed health (five categories defined in increments of 0.2, with respect to SSC) using the ensemble model during the period 1966–2014.

The variation of spatial extent of various watershed health categories with time was also examined. First, the WH index was discretized into five groups in increments of 0.2. Then, the percentages of total area in each of these five discrete groups were calculated for each year. Figure 6 shows the percentage variation of each group over the entire study period. About 15% of the study area had poor watershed health (0.0–0.2), about 40% had watershed health in the range of 0.2 to 0.4, 20% of the area had watershed health between 0.4 to 0.6, 10% of the area under 0.6 to 0.8, and 15% in the range 0.8 to 1.0. The percentage area in the last three groups of relatively medium to good watershed health (0.4–0.6, 0.6–0.8, and 0.8 to 1.0) remained relatively unchanged over the study period. There were small variations for the remaining two, relatively poor watershed health categories (0–0.2, 0.2–0.4), but did not show any long-term trend. The results in Figure 6 correspond to the chosen standard of 30 mg/L for SSC. Mallya et al. [48] note that the percentage areas in each of the five discrete groups is sensitive to the choice of this numerical standard.

#### 4.1.2. Watershed Health for Nutrients

As the study region has dominant agricultural land use, the performances of machine learning models to predict the WH index at ungauged HUC-10 basins with respect to nutrients, namely nitrogen and phosphorus, were also evaluated.

##### Watershed Health for Nitrite + Nitrate

Nitrite + nitrate observations (parameter code: 00631) available at 70 USGS-NAWQA stations were used to evaluate the performance of ML models with respect to nitrogen. As with other water quality constituents, only sporadic data samples were available at these stations. Therefore, daily reconstructed load series of nitrite + nitrate were first obtained following Hoque et al. [8]. Then, using a standard of 10 mg/L [57], the annual series of risk measures reliability, resilience, and vulnerability were calculated and the annual series of WH index was obtained using Equation (6). Similar to SSC, the four machine learning models for WH index were trained and tested (using 80–20% split) using attributes (X) collected for USGS-NAWQA drainage areas and predictions were obtained for ungauged HUC-10 basins (Table 1). For nitrogen and phosphorus analysis, we also included average and total fertilizer sales data as inputs. Therefore, we had a total of 83 explanatory variables in the analysis. The $R^{2}$ values of ML models for WH index (with respect to nitrogen) during training and testing phases, along with the top five explanatory variables according to the RF model are presented in Table 1.

**Figure 7.**
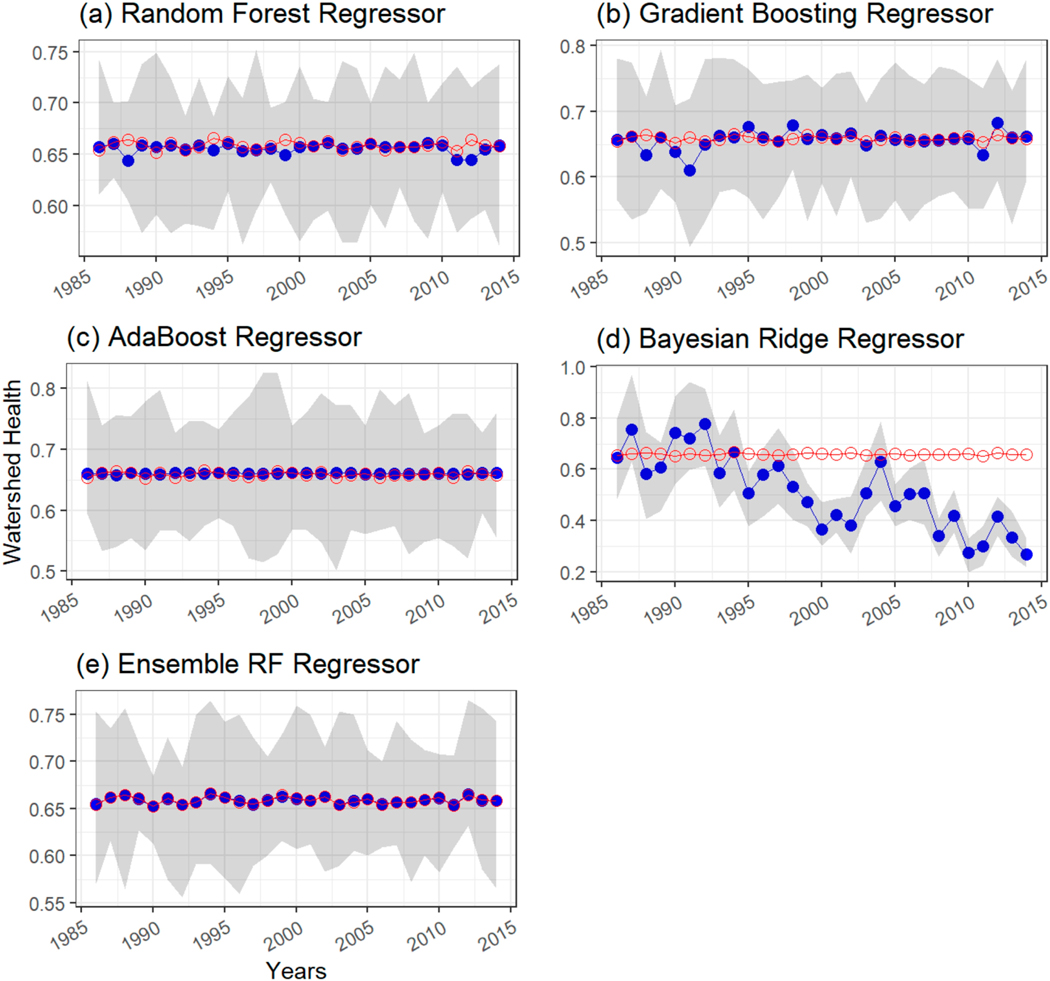
Same as Figure 5. Time series comparison of watershed health (with respect to nitrite + nitrate) estimated from observations as in Mallya et al. [48] at USGS-NAWQA station 04193500 Maumee River at Waterville, OH, which was part of the test set versus ML model predictions using (**a**) random forest regressor, (**b**) gradient boosting regressor, (**c**) AdaBoost regressor, (**d**) Bayesian ridge regressor, and (**e**) ensemble RF regressor.

Figure 7 shows a comparison of time series plots of reference WH (with respect to nitrogen) estimates based on observations and ML and Bayesian ridge regression models’ predictions of WH (with respect to nitrogen) at USGS-NAWQA station 04193500 Maumee River at Waterville, OH, which was part of the test set. A ninety percent prediction interval for each ML model is shown as a grey-shaded region in the plot. WH values predicted by ML models are shown as solid-blue circular markers, whereas reference WH values are shown as red-hollow circular markers. Figure 7a–c indicates that random forest regression, gradient boost regression, and AdaBoost regression models predict annual WH values close to the reference WH values. All reference WH values lie within the 90 percent prediction interval bands of these three ML models. Figure 7d shows that Bayesian ridge regression underpredicts annual WH values and several reference points lie outside the 90 percent prediction interval. The performance of the Bayesian regression model was similar across several stations for nitrogen, therefore this model was excluded from the ensemble. The ensemble model predictions (Figure 7e) were found to better match the reference WH values based on observations compared with individual model predictions (Figure 7a–c).

**Figure 8.**
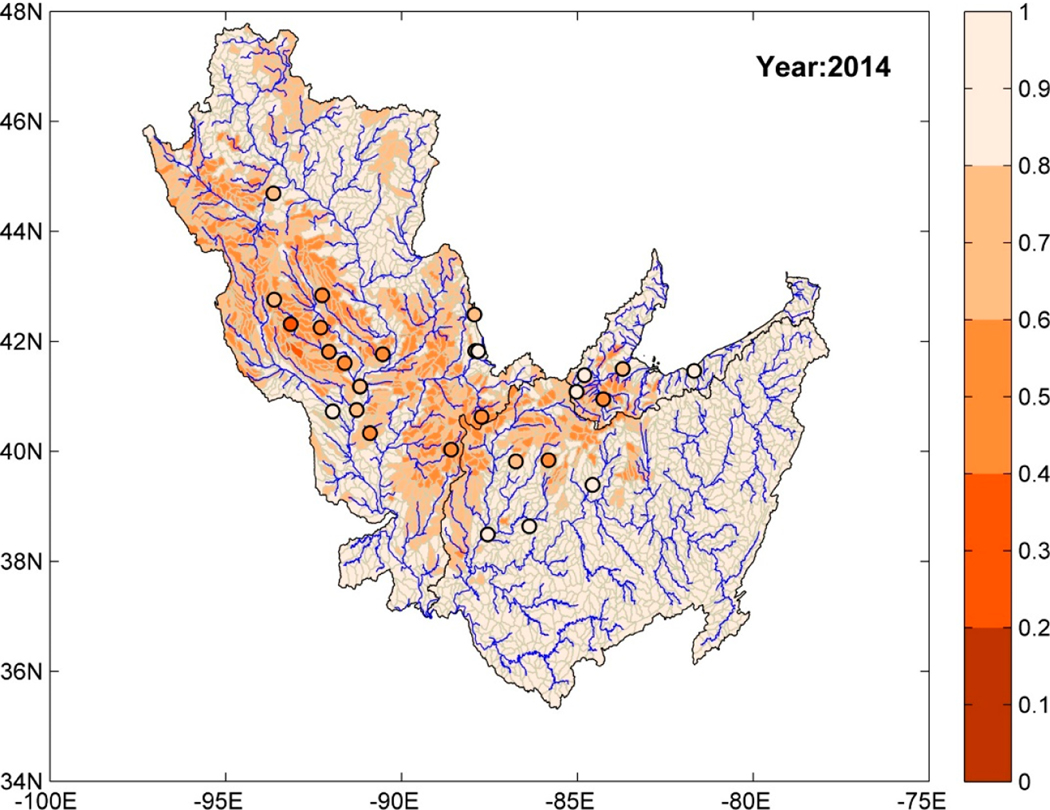
Prediction of watershed health (with respect to nitrite + nitrate) at ungauged HUC-10 basins for the year 2014 using the ensemble model. Circular markers denote the location of USGS-NAWQA stations where nitrite + nitrate measurements were available and are color-coded based on the watershed health for the year 2014.

Since the Bayesian ridge regression model performed poorly in the case of nitrogen (Figure 7d and Table 1) it was not included in the ensemble. The prediction accuracy of an ensemble model is dependent on how well each individual model included in the ensemble performs. Individual model performance statistics such as $R^{2}$ or tests such as the Friedman test and the Nemenyi post hoc test [58] may be used to select models that can be included in the ensemble. Figure 8 shows the watershed health predictions (for nitrite + nitrate) at HUC-10 basins over the study region for the year 2014 using an ensemble model. The circular makers represent USGS-NAWQA stations and they are color-coded to represent reference watershed health values for the year 2014. The regions with poor watershed health (darker color shades) were found to be those with dominant agricultural land use. HUC-10 basins with predominantly forested areas had relatively high watershed health with respect to nitrogen. Similar to the case for SSC, percentages of forest and agricultural land use and longitude were important predictors of watershed health (for nitrite + nitrate) predicted by the RF model (Table 1). Worth noting is the available water storage in the top 25 cm of soil as the second most important predictor. This may underscore the role of plant growth and the uptake of nitrate and subsurface water in nitrogen cycling (nitrification of ammonium nitrogen as a source for nitrite + nitrate and denitrification of nitrate as a sink). As in the case for SSC, the drainage area was identified as an important predictor of WH index, which is not unintuitive since the greater the percentage of the drainage area used for agricultural, the greater the potential for sediment and nutrient supplies.

The variation of spatial extent with time of WH for nitrite + nitrate was also investigated. About 65% of the study area had high watershed health (0.8–1.0), about 20% had watershed health in the range of 0.8 to 0.6, and the remaining portion was in the range 0.4 to 0.6. The percentage areas in each group remained relatively unchanged over the study period. Readers are referred to the Supplementary Information for a detailed discussion of the results for nitrogen.

##### Watershed Health for Orthophosphate

Orthophosphate (parameter code: 00671) values were available at a total of 49 stations over the study region. Following Hoque et al. [8], observed samples were used to reconstruct a continuous daily time series of orthophosphate loads. Using a standard of 0.1 mg/L [57] the annual series of risk measures and WH index were obtained at each station. Table 1 provides a summary of $R^{2}$ values of ML models and the top five explanatory variables of the WH index with respect to orthophosphate.

**Figure 9.**
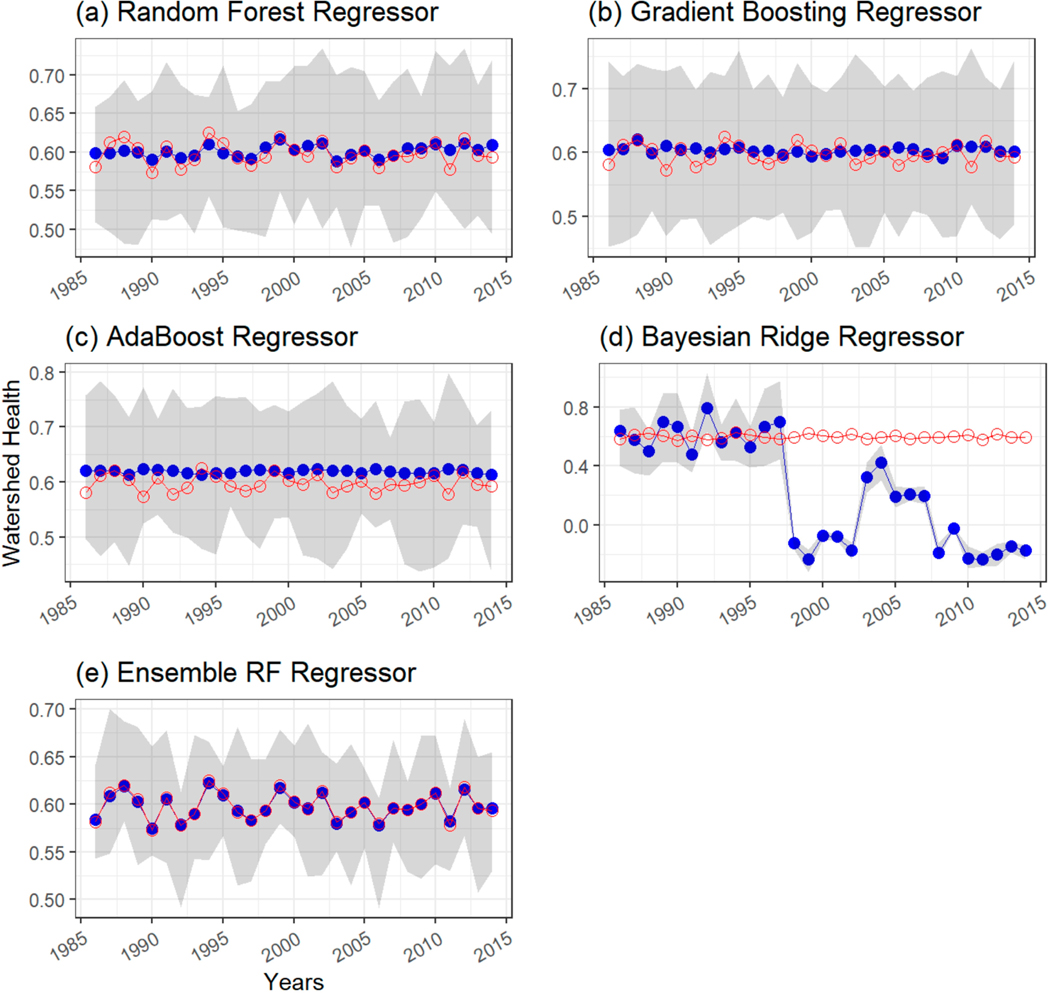
Same as Figure 5. Time series comparison of watershed health (with respect to orthophosphate) estimated from observations as in Mallya et al. [48] at USGS-NAWQA station 04193500 Maumee River at Waterville, OH, which was part of the test set versus ML model predictions using (**a**) random forest regressor, (**b**) gradient boosting regressor, (**c**) AdaBoost regressor, (**d**) Bayesian ridge regressor, and (**e**) ensemble RF regressor.

Figure 9 shows a comparison of time series plots of reference WH (with respect to orthophosphate) estimates based on observations and ML and Bayesian ridge regression models’ predictions of WH at USGS-NAWQA station 04193500, Maumee River at Waterville, OH, which was part of the test set. The grey-shaded regions in the plots denote the ninety percent prediction interval for each ML model. WH values predicted by ML models are shown as solid-blue circular markers, while reference WH values are shown as red-hollow circular markers. Figure 9a,b indicate that random forest regression and gradient boost regression models predict annual WH values close to the reference WH values, whereas the AdaBoost regression model sightly overpredicts WH values (Figure 9c). All reference WH values lie within the 90 percent prediction interval bands of these three ML models. Figure 9d shows that Bayesian ridge regression underpredicts annual WH values at this station and the predicted value is outside the range 0–1. Many reference WH values based on observations lie outside the 90 percent prediction interval (Figure 9d). The performance of the Bayesian regression model was similar across other stations for orthophosphate, therefore this model was excluded when calculating ensemble model predictions. Predictions of WH using the ensemble ML model were found to be close to reference WH values, indicating its robustness (Figure 9e). It was also observed that the width of the 90% prediction intervals were wider in the case of ML models for nutrients compared with SSC, mostly due to a smaller number of training samples in the case of nutrients.

**Figure 10.**
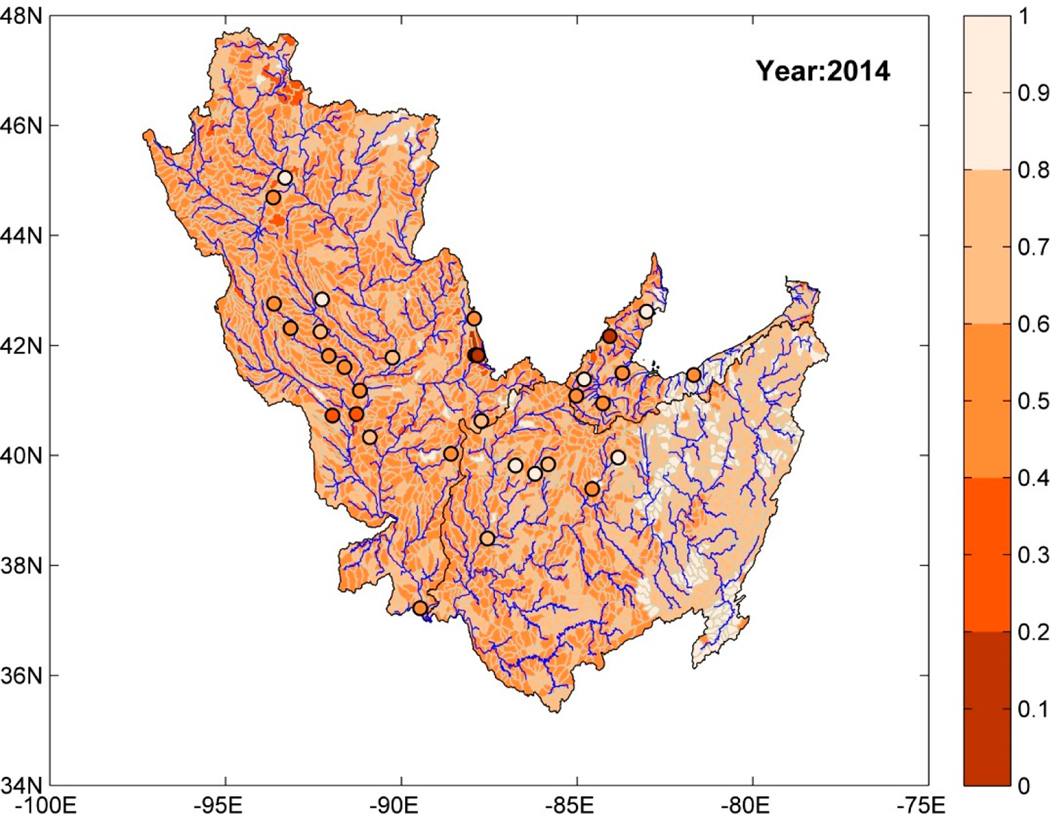
Prediction of watershed health (with respect to orthophosphate) at ungauged HUC-10 basins for the year 2014 using the ensemble model. Circular markers denote the location of USGS-NAWQA stations where orthophosphate measurements were available and are color-coded based on watershed health for the year 2014.

Figure 10 shows the predictions of watershed health over the entire study region for the year 2014. The actual observations (computed values) at individual stations are shown using color-coded circular markers. HUC-10 basins associated with lower stream orders have higher watershed health (with respect to orthophosphate). The regions of highest watershed health in the Ohio River Basin have dominant forest land use. However, the same is not true for the Upper Mississippi River Basin (UMRB); some basins within UMRB that are in the densely forested region have low watershed health. Agriculturally dominant regions were found to have moderate watershed health. The ambiguity in the results may be due to fewer spatially representative sampling stations for orthophosphate, but further investigation is needed to verify this hypothesis given that forest stewardship programs are in place in UMRB.

As for SSC and nitrite + nitrate, forest land-use percentage and longitude were also identified as important predictors of watershed health for orthophosphate for the RF model (Table 1). Average fertilizer sales percentage and areas with hydrologic soil group B were equally important predictors. The former is an indication of the amount of orthophosphate that farmers may have applied over agricultural fields. The moderately well-drained hydrologic soil group B may reflect the importance of shallow subsurface water in regulating and transporting dissolved mineral phosphorous. Worth noting is that tile drains are extensively used in agricultural areas in the upper Midwest. Tile drains may act as bypass pathways, transporting dissolved and colloidal orthophosphate from moderately and well-drained agricultural fields to nearby streams.

Finally, we investigated the variation of spatial extent with time of WH for orthophosphate. Areas with moderate watershed health (0.4–0.6) were dominant with about 60% coverage. About 3% of the total study area had watershed health in the range of 0.2 to 0.4, 60% of the area was in the range of 0.4 to 0.6, 29% of the area was in the range 0.6 to 0.8, and 8% in the range 0.8 to 1.0. The percentage area in the above three groups (0.2–0.4, 0.6–0.8, and 0.8 to 1.0) remained relatively unchanged over the study period. No portion of the study area had poor watershed health (0.0–0.2). There were small variations for the area under moderate watershed health (0.4–0.6), but there was no long-term trend. Readers are referred to the Supplementary Information for a detailed discussion of the results for orthophosphate.

The results indicate that the overall watershed health with respect to orthophosphate is poorer compared to that with nitrogen. This may be because orthophosphate mirrors the sedimentary cycle [59]; this study found that most HUC-10 basins in the study area experience poor to moderate watershed health with respect to SSC (see Figure 6).

## 5. Summary and Conclusions

An ensemble model obtained from four machine learning (ML) models, namely random forest, gradient boosting, AdaBoost, and Bayesian ridge regression, was used in this study to predict recently proposed composite watershed health metric values [48] at ungauged basins with respect to sediments and nutrients. The ML models and their ensemble model were tested in three major river basins, namely the Upper Mississippi River Basin, the Ohio River Basin, and the Maumee River Basin. The ML models require data for watershed attributes, long-term climate, soil properties, land use and land cover, and fertilizer sales to estimate the watershed health index at the ungauged HUC-10 basins. During the training phase, the ML models were trained using data from 80% of the stations in the study region, with a five-fold cross-validation approach. The performance of all ML models was evaluated on an independent test data set that was not used during training (i.e., the remaining 20% of the stations). An ensemble model was then used to combine the results obtained from the individual ML models. To the best of the authors’ knowledge, this is the first such study where machine learning models are used to estimate watershed health using WH metrics [48]. Spatial maps of the watershed health metric values at the HUC-10 basin scale were developed to aid decision makers in identifying critical source areas or hotspots with respect to different water quality constituents. Major conclusions were:

1. For suspended sediment concentration and nitrogen, high watershed health values were often associated with lower order streams and regions with dominant forest land use. Regions with dominant agricultural land use had poor watershed health. Among the predictor variables, land use, geographic position, drainage area, available water storage in soil, hydrologic soil group, fertilizer sales (for nitrogen and orthophosphate), and annual precipitation were found to be significant for the three WQ constituents considered in this study.
2. As a smaller number of stations with phosphorus data were available over the region, the resulting ML models were not robust compared with models developed for SSC and nitrogen. Counter to expectation, even forested watersheds in the UMRB indicated poor WH values with this constituent. More orthophosphate data would be needed at multiple watersheds to not only obtain robust results but to also address the question of attribution to poor (or good) watershed health.
3. Sparse water quality data can be used to predict watershed health and identify impairment source areas in ungauged watersheds.

Individual ML models perform well when there were enough data during the training phase (e.g., SSC). However, when data were limited (e.g., nitrogen and orthophosphate), individual model performance dropped and the development of an ensemble model helped to boost the performance. In this study, the uncertainty in the results of the ML models is presented in the form of prediction intervals. When analysing the 90% prediction intervals at test stations, except for the Bayesian ridge regressor the remaining four ML models performed well. The 90% prediction intervals were found to be wider in the case of ML models for nutrients, which could be attributed to the smaller number of training samples.

The WH metric predictions obtained from the ML models are dependent on the quality of input data. However, in this study we did not analyze the sensitivity of the results to variations in input data values or in the event of missing data. Additionally, if the input data to the ML models are outside the training data range, the output from the ML model would exhibit wider uncertainty.

In this study, the ensemble model performed better than the individual models. As observed in this study, if we have different models designed to predict WH values over an ungauged basin, then each model may produce different values for WH. Further, each individual model will exhibit different performance depending on the type of dataset (SSC, nitrogen, etc.) and the number of training samples available. While one option is to recommend the use of the best performing model [58], the preferred approach in the machine learning community is to use an ensemble approach [60,61]. Generally, to achieve a good ensemble model, each individual model should be as accurate as possible and models should be diverse in their modeling approaches. While there are many strategies to create an ensemble model [62], in this study we have used the outputs from each individual model (random forest, gradient boosting regressor, AdaBoost, etc.) as explanatory variables in a separate random forest (ensemble) model to generate the final WH outputs. However, because the Bayesian ridge regression model performed poorly for nitrogen and orthophosphate (based on R2 values shown in Table 1), it was not used in the ensemble model. Only the outputs from three ML models (i.e., random forest, AdaBoost, and gradient boost) were used as input to the ensemble model for nitrogen and orthophosphate.

The risk-based methodology, machine learning modeling framework, and insights learned from the application to three major Midwest river basins can be applied to different water quality parameters (e.g., pesticides, pathogens, etc.) and are transferable to similar watersheds in the US and other parts of the world. Such a screening-level data-driven approach can inform resource managers and environmental decision makers where to focus their resources for targeted distributed watershed modeling and localized evaluation of best management practices.

## Supplementary Material

Supplementary Material

## Figures and Tables

**Table 1.** Goodness of fit measures of ML models and top five predictors of watershed health. The coefficient of determination $R^{2}$ for the training set is the average value obtained during 5-fold cross validation, while the value given between the parentheses is the $R^{2}$ value for the test set.

	SSC	Nitrite + Nitrate	Orthophosphate
**Number of stations**	151	70	49
**US EPA standard (mg/L)**	30	10	0.1
$R^{2}$ **training (testing)**			
Random forest	0.98 (0.95)	0.98 (0.81)	0.99 (0.26)
Gradient boosting	0.99 (0.94)	0.99 (0.84)	0.98 (0.57)
AdaBoost	0.87 (0.84)	0.99 (0.88)	0.94 (0.32)
Bayesian ridge	0.75 (0.68)	0.75 (−1.31)	0.83 (−22)
Ensemble	0.98 (0.98)	0.97 (0.98)	0.98 (0.99)
**Top 5 predictors**	Forest land-use percentage	Agricultural land-use percentage	Water land-use percentage
	Water land-use percentage	Available water storage in top 25 cm of soil	Average fertilizer sales
	Longitude	Drainage area	Longitude
	Agricultural land-use percentage	Forest land-use percentage	Forest land-use percentage
	Drainage area	Longitude	Percentage area with hydrologic

## Data Availability

Refer to Supplementary Information for detailed information on datasets used in this study.
