# Supplementary material for: A Machine Learning Approach to Predict Watershed Health Indices for Sediments and Nutrients at Ungauged Basins
Source: Water (Basel). Author manuscript; Available in PMC 2023 Jun 12. (PMC10259765; doi:10.3390/w15030586)
Supplement: Supplementary Material [file NIHMS1885786-supplement-Supplementary_Material.pdf]

Supplementary Information for:

A Machine Learning Approach to Predict Watershed Health Indices for Sediments and Nutrients at Ungauged Basins

Ganeshchandra Mallya [1], Mohamed M. Hantush [2,*] and Rao S. Govindaraju [1]

[1] Lyles School of Civil Engineering, Purdue University, West Lafayette, IN 47907, USA; ganeshchandra.mallya@gmail.com (G.M.); govind@purdue.edu (R.S.G.)
[2] U.S. EPA Center for Environmental Solutions and Emergency Response, 26 West Martin Luther King Dr., Cincinnati, OH 45268, USA
* Correspondence: hantush.mohamed@epa.gov

Number of pages: 28

Number of figures: 20

S.1 Datasets

S.1.1 Streamflow and water quality

This study primarily focused on risk-based assessment of watershed health (Mallya et al., 2018) with respect to the following water quality constituents - suspended sediment concentration (SSC), nitrogen, and phosphorus. To perform a risk-based analysis, we require a continuous time series of water quality. But these are not often available, and have to be reconstructed by using continuous time series of streamflow observations as surrogate information (Hoque et al., 2012; Runkel et al., 2004). Water quality data available from United States Geological Survey – National Water Quality Assessment (USGS-NAWQA) program were used in this study. A total of 214 stations with a minimum of 30 observations for any of the three water quality constituents were available over the study area. The limit on the minimum number of observations ensures that the reconstruction process is robust. Among the 214 stations, suspended sediment concentration (SSC) data were available at 151 stations; nitrite + nitrate data at 70 stations, and orthophosphate data at 49 stations. In addition to water quality data, these stations also had records of continuous daily streamflow. The geographic location of these stations is denoted as green circular markers in Figure 1. The period of analysis extends from 1965 to 2014, and was chosen based on availability of streamflow data at these stations.

S.1.2 Geographic data

The Hydrologic Unit Code 10 (HUC-10) boundaries were obtained from USGS National Hydrography Dataset (NHD). The HUC-10 boundaries have an average area of about 580 square kilometers, and are representative of hydrologic processes at watershed-scale. A total of 2354 HUC-10 basins are available over the study area and are shown as grey polygons in Figure 1. The NHD dataset also contains vector shapefiles that represents the stream network over the study area,

and these were used to burn-in streams during watershed delineation for USGS-NAWQA stations. The delineated watersheds and HUC-10 boundaries were used for quantifying watershed attributes and other predictor variables used as inputs for ML models.

S.1.3 Land use and Land cover data

The 2011 National Land Cover Database (NLCD) which is based on a decision-tree classification of Landsat satellite data was used in this study. The spatial resolution of the data is 30 meters. The original data consists of 20 classes of land use. These were then combined to represent four broad classes - agriculture, forests, urban, and water. Figure S1 shows the land use map comprising of these four broad classes.

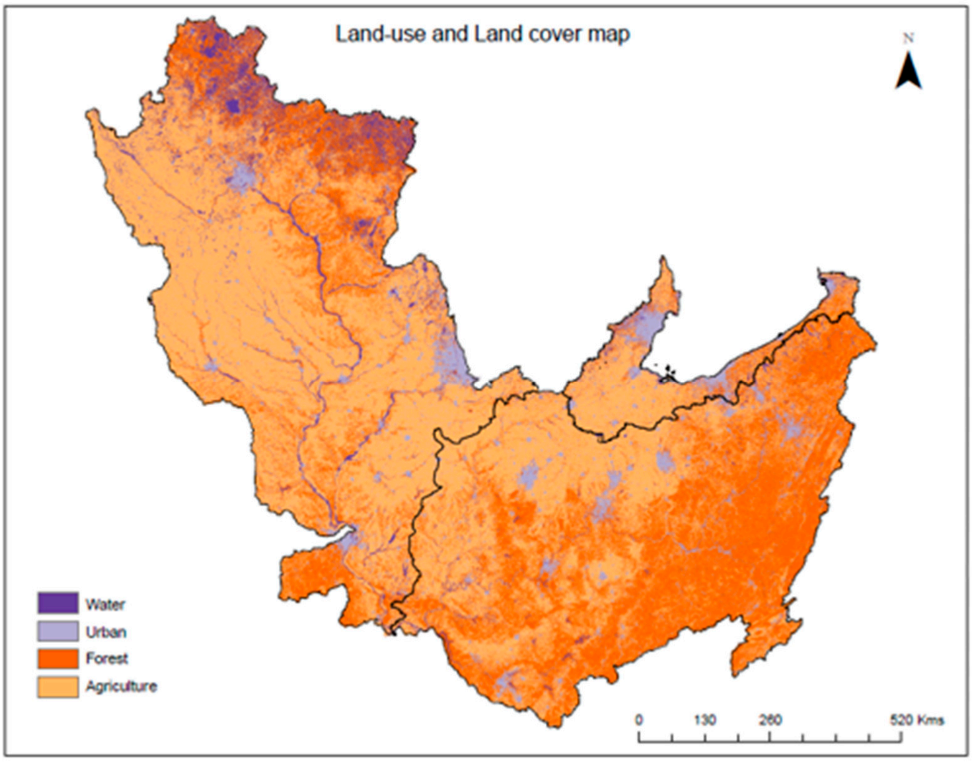

Figure S1: NLCD 2011 land use map over the study area. The original land use classes have been reclassified into four broad classes – agriculture, forest, urban, and water. See Figure S2 for percentage distribution of these broad classes in each river basin.

Using GIS, the land use percentages corresponding to each of the four broad cases were computed for each HUC-10 basin in UMRB, ORB, and MRB. As seen in Figure S2a, approximately 83% of land in UMRB belongs to forest and agriculture land use type, 9% is urban/residential, and about 8% comprise of water bodies. As in case of UMRB, forest and agriculture land use classes are predominant in ORB, constituting about 88% of the total drainage area (see Figure S2b). Urban/residential land use is 10%, and water bodies cover around 2% of drainage area in ORB. In MRB (Figure S2c) 70% of the area is under agriculture and forest land use, 24% area has urban land use and the remaining 6% are water bodies.

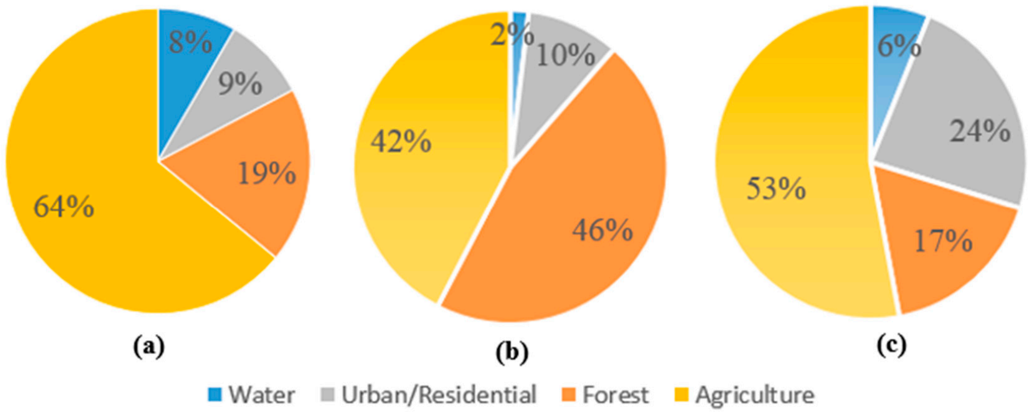

Figure S2: Percentage of four broad land use classes – agriculture, forest, urban, and water in a) UMRB, b) ORB, and c) MRB, respectively.

S.1.4 Soil data

Soil Survey Geographic Database (SSURGO) was used to extract soil properties for the study area. Among the several soil properties that were available in the SSURGO database, we have used *hydrologic soil group* and *available water storage* (AWS) in top 25 cm of soil in this study. Hydrologic group A soils are mainly composed of sand, loamy sand or sandy loam types of soils. They have high infiltration rates and low runoff potential. Hydrologic group B soil are mostly silt loam or loam and have moderate infiltration rates. Hydrologic group C soils are sandy clay loam with low infiltration rates, and group D soils are clay loam, silty clay loam, silty clay, sandy clay and clay soil with very low infiltration rates and high runoff potential. Figure S3 shows the percentage distribution of each soil group in each HUC-10 basin within the study area. While large parts of UMRB, southern portions of ORB, and western parts of MRB are dominated by soils belonging to hydrologic soil group B, northern and eastern portions of ORB and southern parts of UMRB and MRB have high percentage of soils that belong to hydrologic soil group C. The eastern parts of MRB belong to hydrologic soil group D.

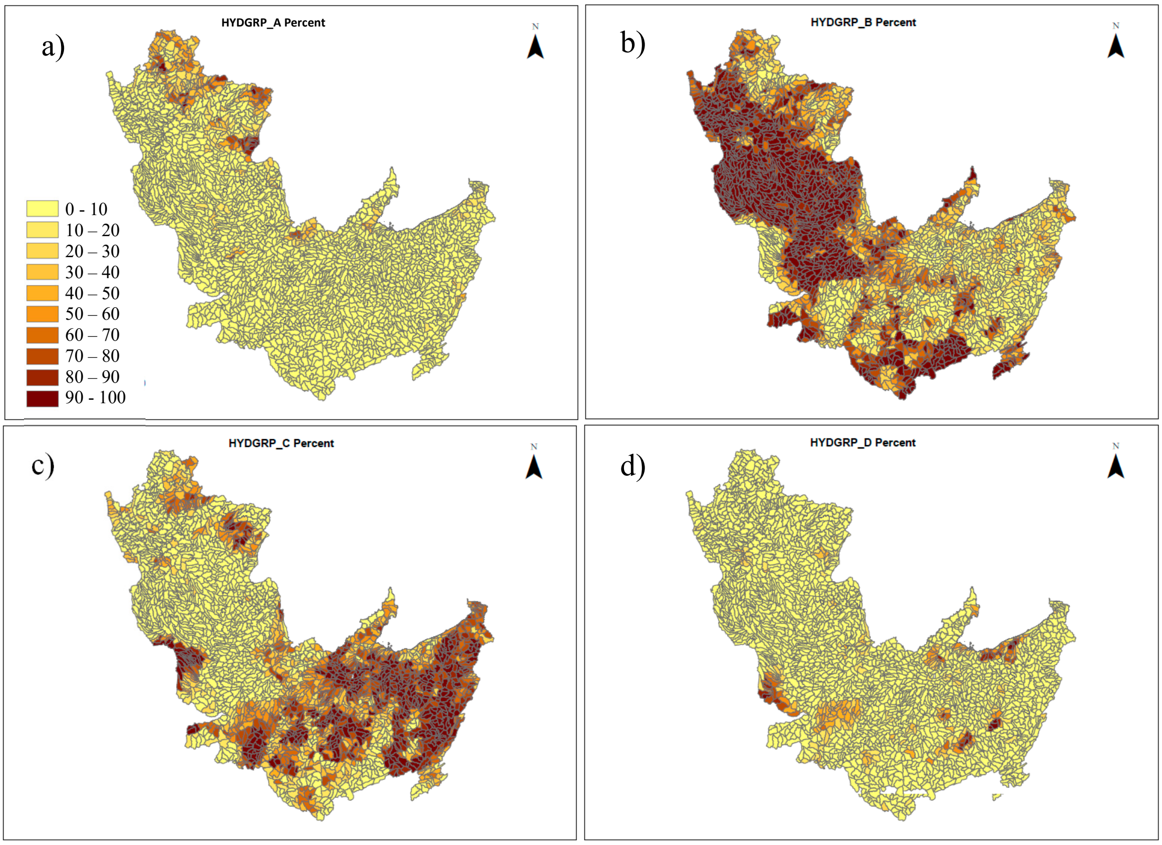

Figure S3: Percentage of each hydrologic soil group belonging to - a) Group A, b) Group B, c) Group C, and d) Group D.

Figure S4 shows the average available water storage (cm/cm) in top 25 cm of the soil. This measure denotes the water holding capacity in the top soil that is available for evapotranspiration and other hydrologic processes. The areas with high values of available water storage are predominantly of agricultural land use and those with low values are predominantly forested (see Figure S1).

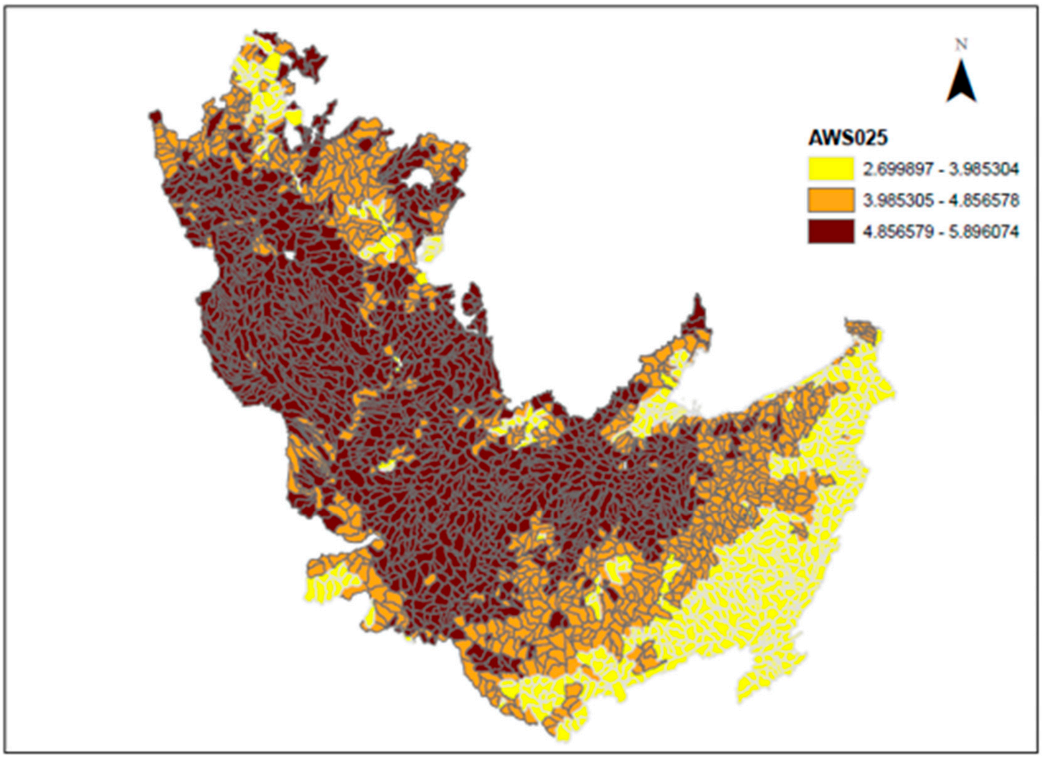

Figure S4: Average available water storage in top 25 cm of the soil (cm/cm).

S.1.5 Long-term climate data

Daily precipitation (PRCP), minimum and maximum temperature (TMIN and TMAX) data were collected over the study area using the Historical Climate Network (HCN) stations available at National Centers for Environmental Information (NCEI) website. Stations with a minimum record of 30 years were considered in this study. Daily data were converted to monthly, seasonal, and annual time scales. Thiessen polygons were constructed for all the HCN stations, and then the area weighted precipitation and minimum- and maximum-temperatures were computed for each HUC-10 basin, as well as for areas draining to USGS-NAWQA stations. As part of exploratory data analysis (EDA) a Mann-Kendall (MK) trend test with $\alpha = 0.05$ was performed to identify HCN

stations that have statistically significant trend in these climate variables at monthly, seasonal, and annual scales, during the study period.

Figure S5 shows the average annual precipitation total at each HCN station over the study area. Locations exhibiting statistically significant positive trends in annual total precipitation (increasing over time) are denoted using red colored square markers, while those exhibiting significant negative trends are denoted as blue colored square markers. The majority of the stations in the study area do not have statistically significant trends (982 out of 1167 stations in UMRB, 1600 out of 1757 stations in ORB, and 204 out of 231 stations in MRB). However, the number of stations with significant positive trends (177 out of 1167 stations in UMRB, 129 out of 1757 stations in ORB, and 24 out of 231 stations in MRB) outnumber those with negative trends (8 out of 1167 stations in UMRB, 28 out of 1757 stations in ORB, and 3 out of 231 stations in MRB). This suggests an overall increase in total annual precipitation during the study period over this region.

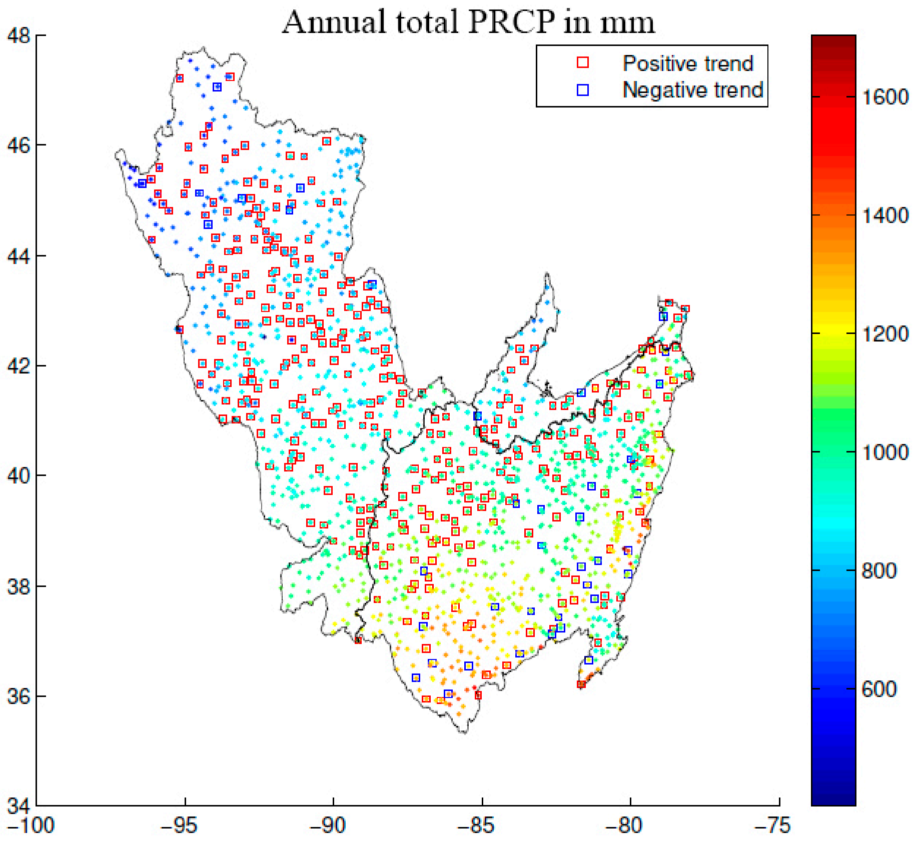

Figure S5: Average annual total precipitation (in mm) at each HCN station (shown as points) over the study area. Stations with statistically significant increasing (positive) and decreasing (negative) trends in annual total precipitation according to MK trend test are also highlighted using red and blue square markers, respectively.

Figure S6a-b shows the mean magnitudes of annual arithmetic average of daily maximum temperature and minimum temperature averages recorded at HCN stations, respectively. As in the case of annual total precipitation, stations with statistically significant positive and negative trends in average maximum or minimum temperatures are denoted as red and blue square markers, respectively. The percentage of HCN stations with significant trends in annual average temperature

(both maximum and minimum) are more in number over ORB, compared to UMRB and MRB. For mean annual average of maximum daily temperature (Figure S 6a), the number of stations exhibiting significant negative trends (71 out of 756 stations in UMRB, 144 out of 1078 stations in ORB, and 13 out of 169 stations in MRB) outnumber those with positive trends (37 out of 756 stations in UMRB, 57 out of 1078 stations in ORB, and 10 out of 169 stations in MRB). This suggests an overall decrease in maximum temperatures during the study period over this region. However, the opposite is true for annual average minimum temperature (Figure S 6b). The number of stations with significant positive trends (145 out of 753 stations in UMRB, 137 out of 1065 stations in ORB, and 27 out of 166 stations in MRB) outnumber those with negative trends (26 out of 753 stations in UMRB, 69 out of 1065 stations in ORB, and 4 out of 166 stations in MRB), indicating overall increase in minimum temperatures in the region during the study period.

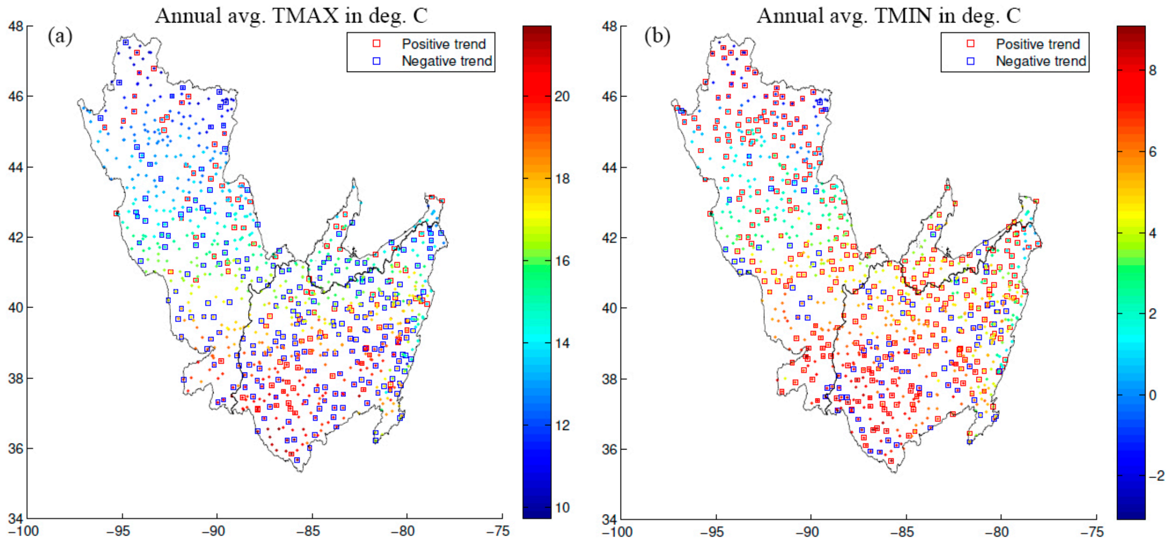

Figure S6: Mean annual average a) maximum and b) minimum daily temperatures in degree Celsius at HCN stations (shown as points) over the study area. Stations with statistically significant increasing (positive) and decreasing (negative) trends in annual average maximum and minimum

temperatures according to MK trend test are also highlighted using red and blue square markers, respectively.

S.1.6 Fertilizer sales data

As the study region has dominant agricultural land use, fertilizer sales data available from United States Department of Agriculture's National Agricultural Statistics Service (USDA-NASS) were used to account for the influence of application of fertilizers on water quality loads and therefore on watershed health with respect to nutrients. The sales data (in million-dollar amounts) were available for each county for the years 1997, 2002, 2007, and 2012. The values of other years of the study period were filled using these available values. For simplicity, years 1966 to 1996 were assigned fertilizer sales data available for 1997. Similarly, years 1998 to 2001 were assigned fertilizer sales data available for 2002, and so on.

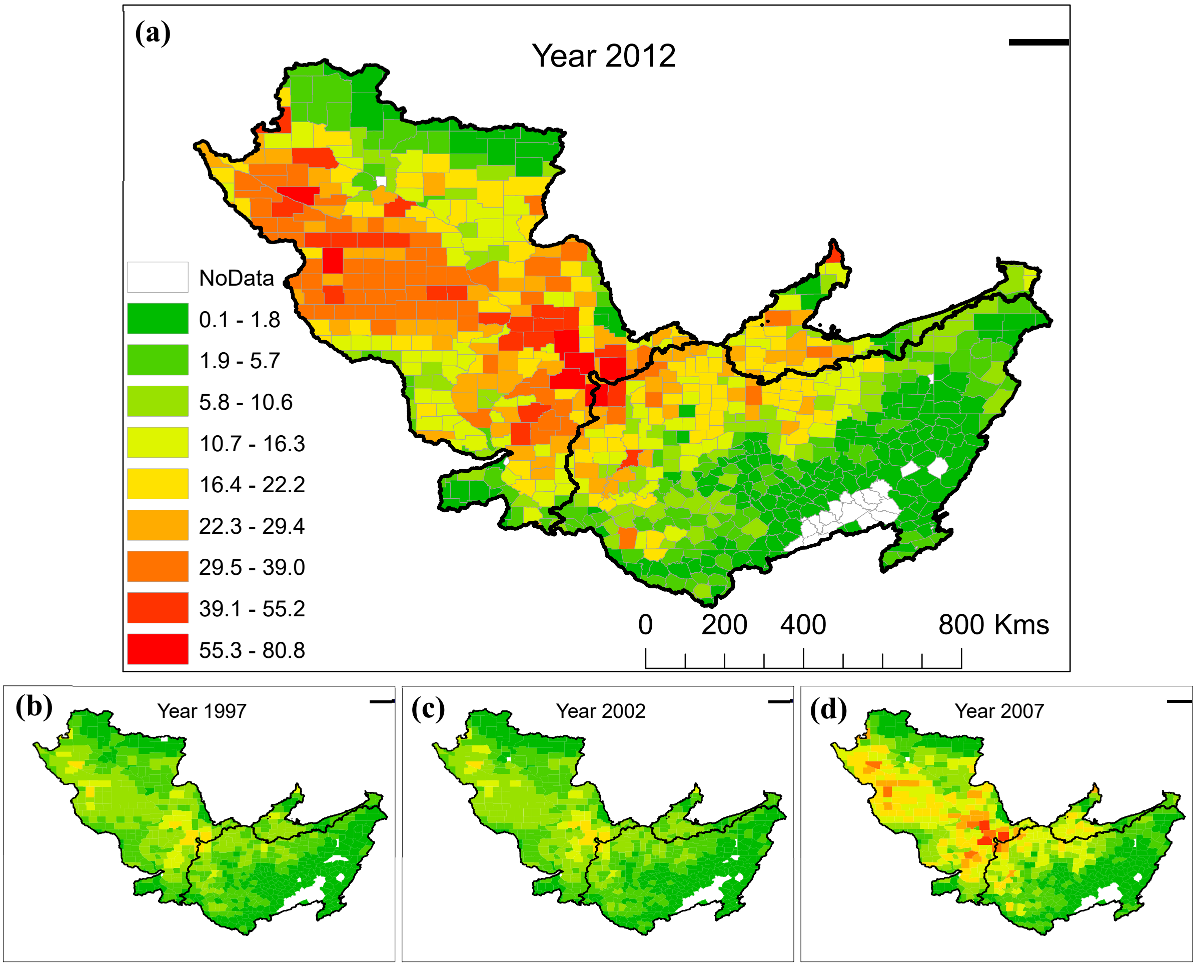

Figure S7: County level fertilizer sales (in million dollars) over the study area for a) 2012, b) 1997, c) 2002, and d) 2007.

Figure S7 shows the county level fertilizer sales data for four time points - 1997, 2002, 2007, and 2012, over the study region. The counties are color coded based on legend shown in Figure S 7a. Fertilizer sales (in million dollars) have been steadily increasing over the period of analysis, and

regions with high sales correspond well with areas having dominant agricultural land use (see Figure S1).

S.1.7 Watershed attributes

Using digital elevation model data (DEM - 30m resolution) drainage areas were delineated for each USGS-NAWQA station. These, along with HUC-10 basins and NHD stream shapefiles, were used to calculate the following watershed attributes - drainage areas, slope, and stream order. Latitude and longitude information were also recorded.

S.2 Additional Results

S.2.1 Suspended Sediments

Random Forest Regression Model – Testing phase

Watershed attributes (X) from 20% of the stations (belonging to the test set following 80:20 split) were used as inputs to the trained RF model, and the outputs (y_{pred}) were compared with reference risk values (computed independently following Mallya et al.(2018)). Figure S 8 shows a scatter plot of reference WH versus predicted annual watershed health values for the entire study period (1966-2014) at all test stations combined. The coefficient of determination R^2 value of 0.95 was obtained on the test set. Though the R^2 statistic is high, there is significant scatter about the best-fit (1:1) line.

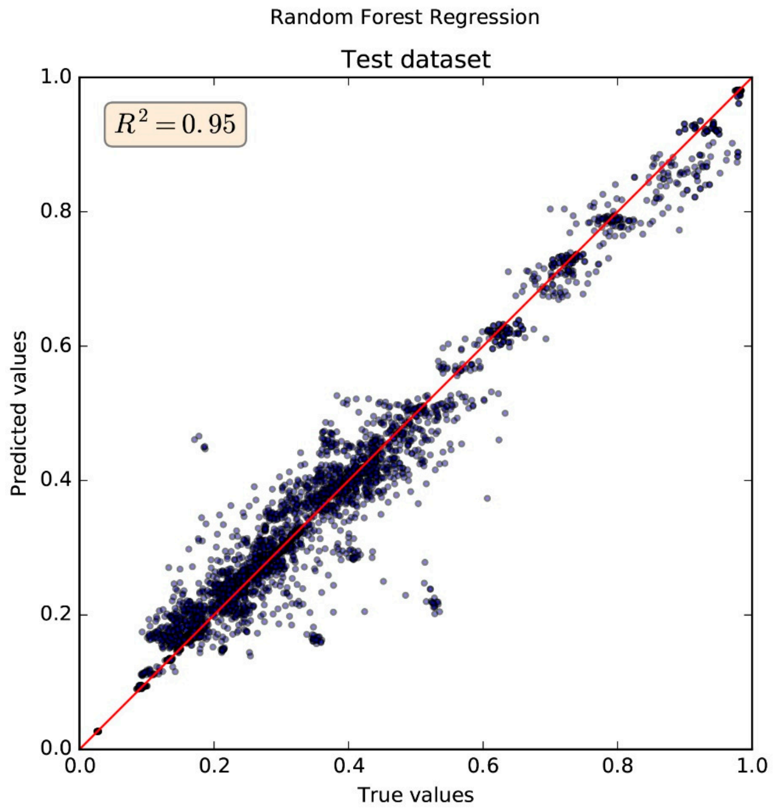

Figure S8: Scatter plot of reference WH versus predicted watershed health (with respect to SSC) using a random forest model at USGS-NAWQA stations that were used as test set. The values for each year during the study period (1966-2014) are shown.

Similar results were obtained when the random forest model was trained on other risk measures. Figure S9a-c shows the scatter plot of reference versus predicted values of reliability, resilience, and vulnerability of SSC, respectively. As in the case of watershed health (see Figure S8), the results shown here are for the test set. The R^2 values for all three risk measures were high but there was scatter around the best fit line, indicating under- or over-prediction for some cases.

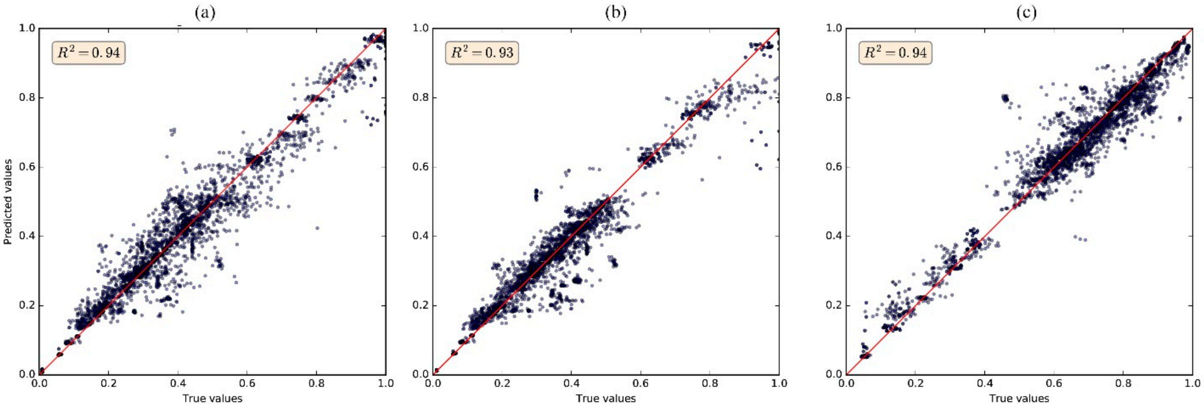

Figure S9: Scatter plot of reference (computed independently following Mallya et al. (2018)) versus predicted (a) reliability, (b) resilience (c) vulnerability (with respect to SSC) using a random forest model at USGS-NAWQA stations that were used as test set. The values for each year during the study period (1966-2014) are shown.

<u>Other regression models – Testing phase:</u>

In addition to using a random forest regression model, we also considered gradient boosting, adaptive boosting (AdaBoost), and Bayesian ridge regression models for predicting risk measures at ungauged HUC-10 basins. The scatter plots of reference WH versus predicted watershed health values (with respect to SSC) on the test dataset according to these regression models are shown in Figure S 10. The gradient boosting regression model yields a R^2 value of 0.94 (Figure S10a), which is comparable to that obtained from the random forest model (see Figure S8). While most points lie along the best fit line, there is considerable scatter indicating lack of strong prediction power. Figure S10b compares the predictions from AdaBoost regression with reference watershed health values. The points do not lie on the best fit line, indicating a relatively poor model fit. The

R^2 value for this model was 0.84. The Bayesian ridge regression (Figure S 10c) had the worst performance among the four models used in the study, with R^2 value of 0.68.

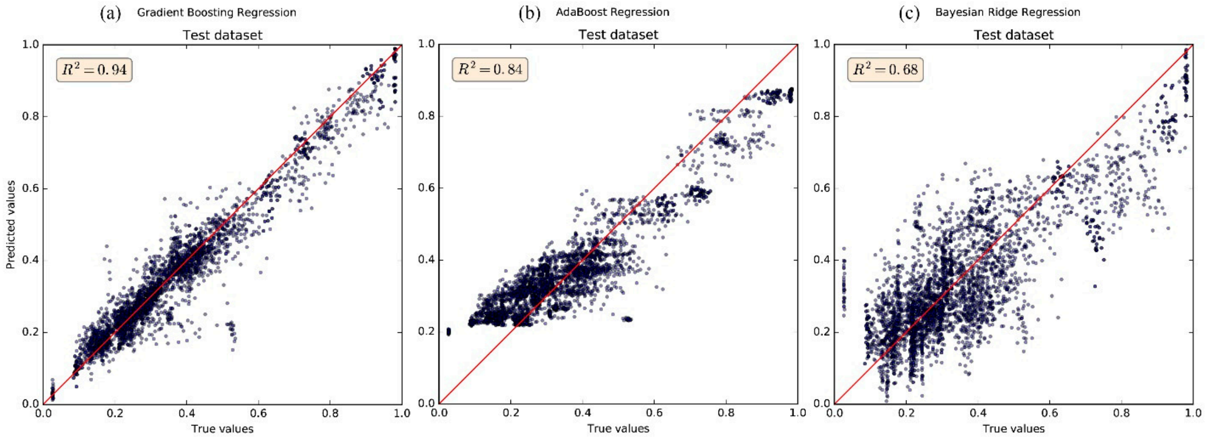

Figure S10: Scatter plot of reference WH versus predicted watershed health (with respect to SSC) using (a) gradient boosting regression, (b) AdaBoost regression, and (c) Bayesian ridge regression at USGS-NAWQA stations that were used as test set. The values for each year during the study period (1966-2014) are shown.

Ensemble model – Testing phase

The WH outputs (from the training phase, i.e., 80% of USGS-NAWQA stations) from the four ML models discussed in this study were used as explanatory variables (X) in a separate random forest model with 50 decision trees. The target variable (y) for this model was still the annual series of *reference* WH measures as used before. The training and testing were performed as described before using 80-20% split of stations. The trained ensemble (random forest) model was then evaluated on the test set. Figure S 11 shows the comparison of reference WH versus predicted WH values over the entire test dataset (20% stations and output for the period 1966-2014). The

points in the scatter plot are tightly bound along the best fit line, with only few instances of significant scatter. The R^2 value was 0.98 on the test dataset.

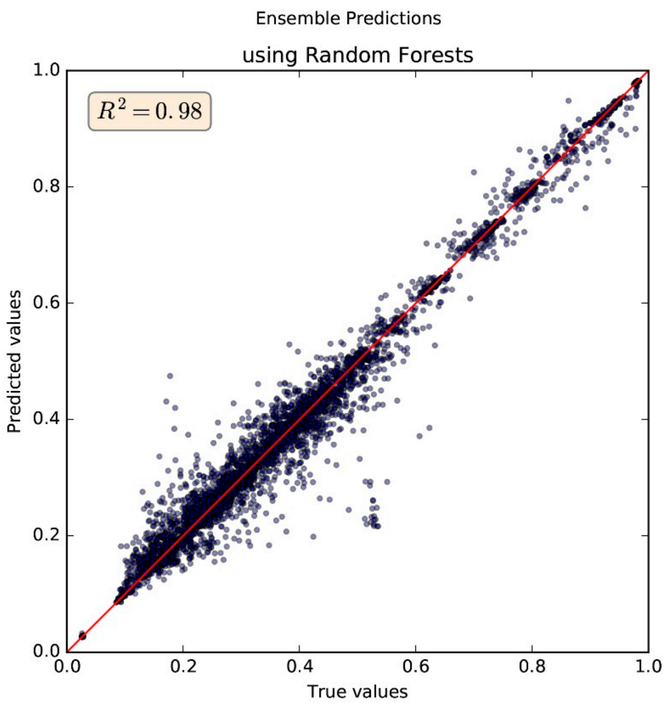

Figure S11: Scatter plot of reference WH values versus ensemble model predictions of watershed health (with respect to SSC) at USGS-NAWQA stations that were used as test set. The values for each year during the study period (1966-2014) are shown.

Ensemble model – Prediction at ungauged HUC-10 basins

Using WH predictions from individual ML models as inputs to the trained ensemble model, we can obtain predictions of WH over ungauged HUC-10 basins. The spatial map of ensemble-predicted watershed health is shown in Figure S 12. The results are for the year 2014. The circular markers denote the geographic location of USGS-NAWQA stations that formed part of training and testing dataset. The HUC-10 basins and stations are color coded similar to Figure 4. The HUC-

10 basins with high watershed health (light shades) belong mostly to the regions with dominant forest land use (refer Figure S1) and those with low watershed health (darker color shades) are in regions with dominant agricultural land use. These results are generally similar to those predicted using the Random Forest model (see Figure 4).

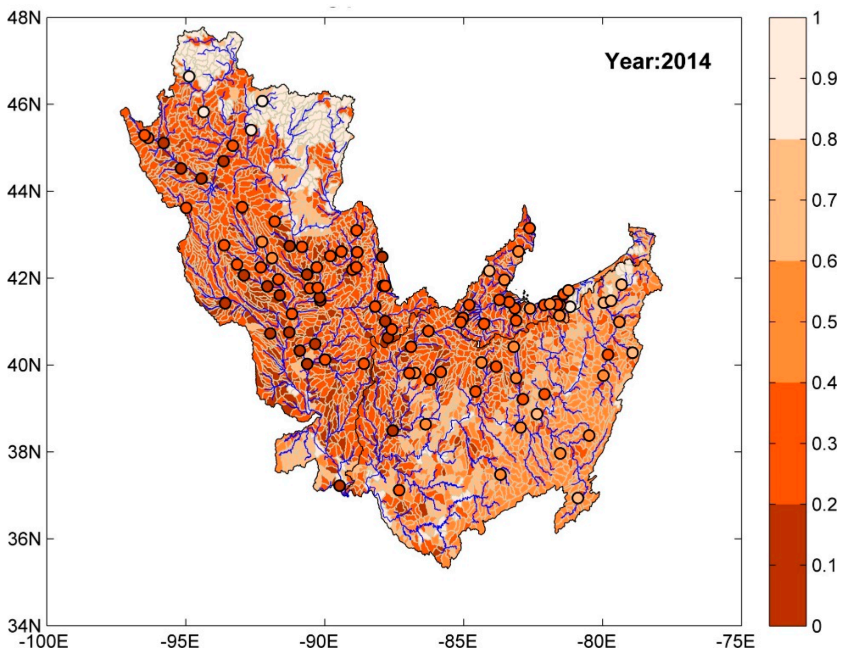

Figure S12: Prediction of watershed health (with respect to SSC) at ungauged HUC-10 basins for the year 2014 using the ensemble model. Circular markers denote the location of USGS-NAWQA stations where SSC measurements were available and are color coded based on watershed health for year 2014.

S.2.2 Watershed health for Nitrite + Nitrate

As the study region has dominant agricultural land use, we evaluated risk measures at ungauged HUC-10 basins with respect to Nitrogen and Phosphorus. In this section, we will take a look at risk evaluations with respect to Nitrogen. We used Nitrite + Nitrate observations (parameter code:

00631) available at 70 USGS-NAWQA stations for this purpose. As with other water quality constituents, only sporadic data samples were available at these stations. Therefore, daily reconstructed series of Nitrite + Nitrate were first obtained following Hoque et al. (2012). Then using a standard of 10 mg/L (US EPA, 1986), annual series of risk measures, such as reliability, resilience, vulnerability and watershed health, were obtained using Equations (1)-(5). The computed risk measures' values are conveniently referred to as reference values. Then for each individual risk measure (y) and similar to SSC, a machine learning model was trained and tested (using 80%-20% split) using attributes (X) collected for USGS-NAWQA drainage areas, and predictions were obtained for ungauged HUC-10 basins. For nitrogen and phosphorus analysis we also included average and total fertilizer sales data as inputs. Therefore, we had a total of 83 explanatory variables in the analysis.

Performances of ML models – Testing phase

Figure S13a-d shows the performance of the four regression models used in this study in predicting watershed health measure (with respect to Nitrite + Nitrate) on the test dataset. Figure S 13a shows the results of random forest model, which had a R^2 value of 0.81. When compared to Figure S 8, the scatter plots for nitrogen (Figure S 13) contains smaller number of data points as there are only 14 stations in the test set (out of total 70 stations). The R^2 values and scatter in reference watershed health values versus predicted watershed health values for gradient boosting, AdaBoost, and Bayesian ridge regression models are shown in Figure S 13b-d. The AdaBoost regression had the highest R^2 value of 0.88, while the Bayesian ridge regression model showed poor performance ($R^2 = -1.31$).

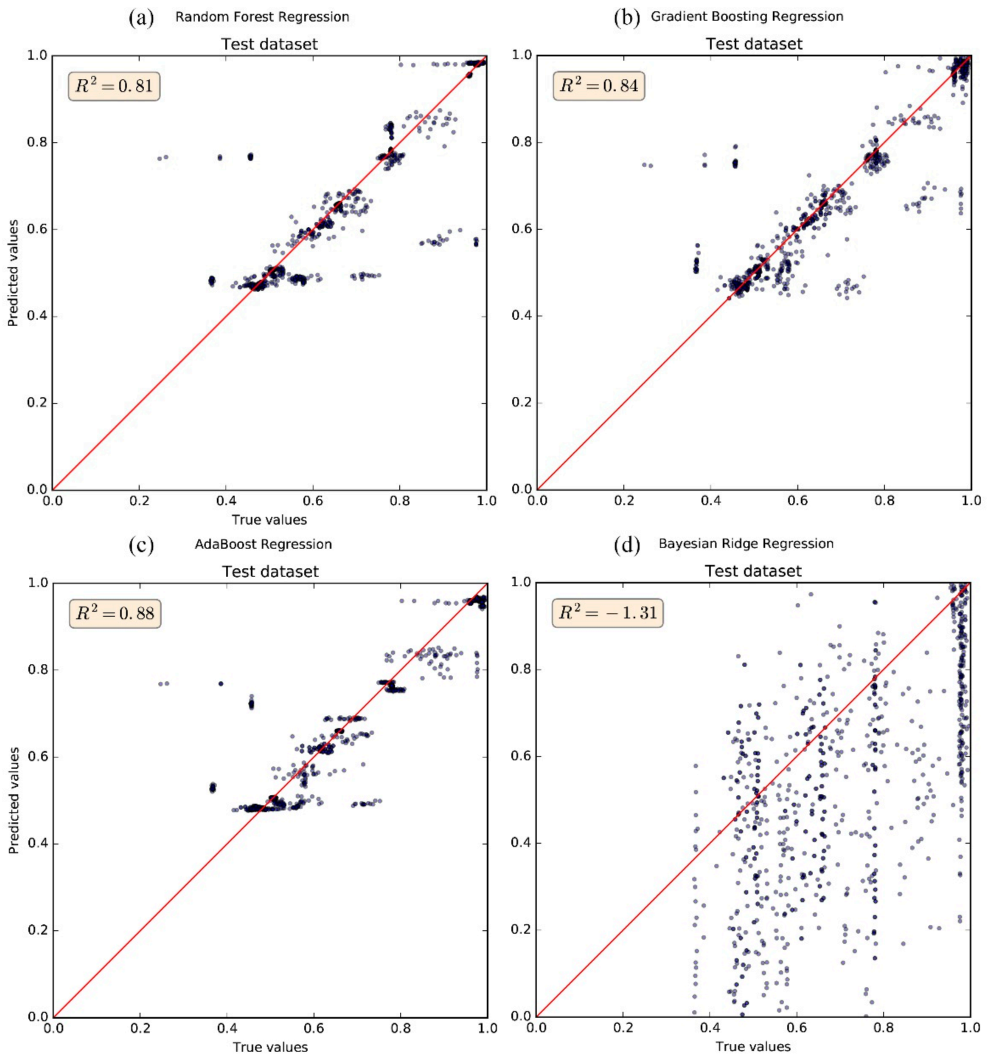

Figure S13: Scatter plot of reference WH versus predicted watershed health (with respect to Nitrite+Nitrate) using (a) random forest regression, (b) gradient boosting regression, (c) AdaBoost regression, and (d) Bayesian ridge regression at USGS-NAWQA stations that were used as test set. The values for each year during the study period (1966-2014) are shown.

<u>Important explanatory variables – Random forest model</u>

Figure S14 shows the top 15 out of 83 variables that were important in explaining watershed health with respect to Nitrite + Nitrate during the training phase of random forest model. Results indicate that percentage of area under agricultural and forest land use, stream order, longitude, available water storage were the most important. Average fertilizer sales data was also among the top 15 important variables.

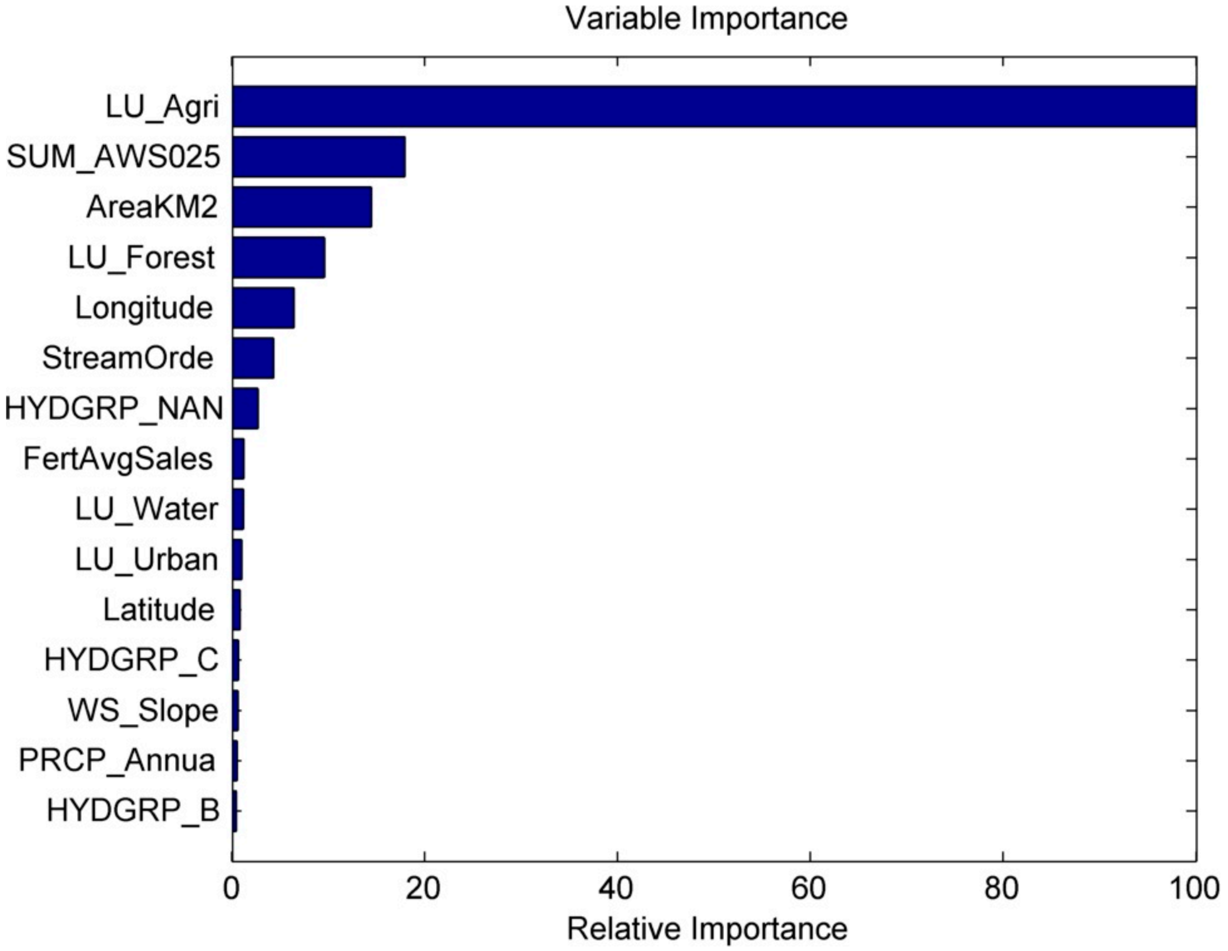

Figure S14: Variable importance for top 15 out of 83 explanatory variables according to random forest model trained on watershed health (with respect to Nitrite + Nitrate) at USGS-NAWQA stations.

Ensemble model – Testing phase

The outputs from the individual models (from the training phase) were then used as explanatory variables (X) in a separate random forest model with 50 decision trees. The target variable (y) was still reference (i.e., computed from observed WQ data) risk measures at USGS-NAWQA stations. The ensemble model was trained using 5-fold CV over 80% of the stations, and then tested over remaining 20% of the stations. Figure S 15 shows the scatter plot of reference WH versus ensemble model predictions of watershed health (for Nitrite + Nitrate) over test stations. The R^2 value was found to be 0.98 over the test set. Most predictions lie on the best fit line, with considerably fewer number of instances with poor predictions.

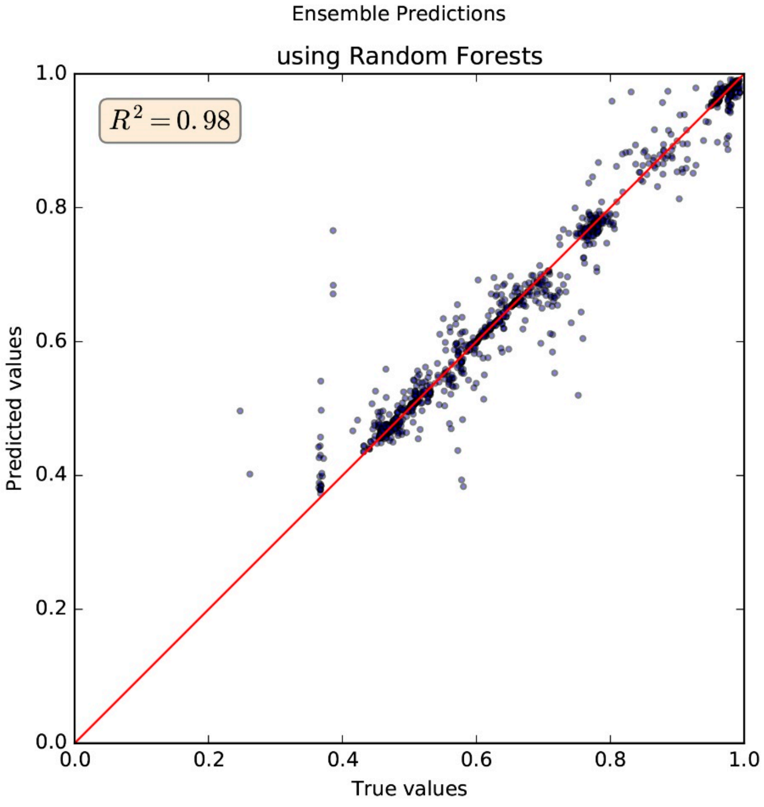

Figure S15: Scatter plot of reference values of WH versus ensemble model predictions of watershed health (with respect to Nitrite+Nitrate) at USGS-NAWQA stations that were used as test set. The values for each year during the study period (1966-2014) are shown.

<u>Variation of spatial extent of WH measure with time</u>

The trained ensemble model was then used to obtain predictions of WH measures over ungauged HUC-10 basins using individual ML model predictions as the input. As in the case of SSC, we also investigated the variation of spatial extent with time of WH measure for Nitrite + Nitrate. Figure S16 in particular shows their annual variations. First, the risk measures were discretized into five groups in increments of 0.2 (also denoted by different color shading). Then, the percentage of total area in each of these five discrete groups were calculated for each year. Figure S16 shows the percentage variation of each group over the entire study period. About 65% of the study area had high watershed health (0.8-1.0), about 20% had watershed health in the range of 0.8 to 0.6, and the remaining portion were in the range 0.4 to 0.6. The percentage areas in each group remained relatively unchanged over the study period. The results in Figure S16 correspond to the chosen standard of 10 mg/L for Nitrite + Nitrate. We expect the distribution of areas in the five discrete groups to be sensitive to the choice of the standard, but we expect the no-trend behavior to hold.

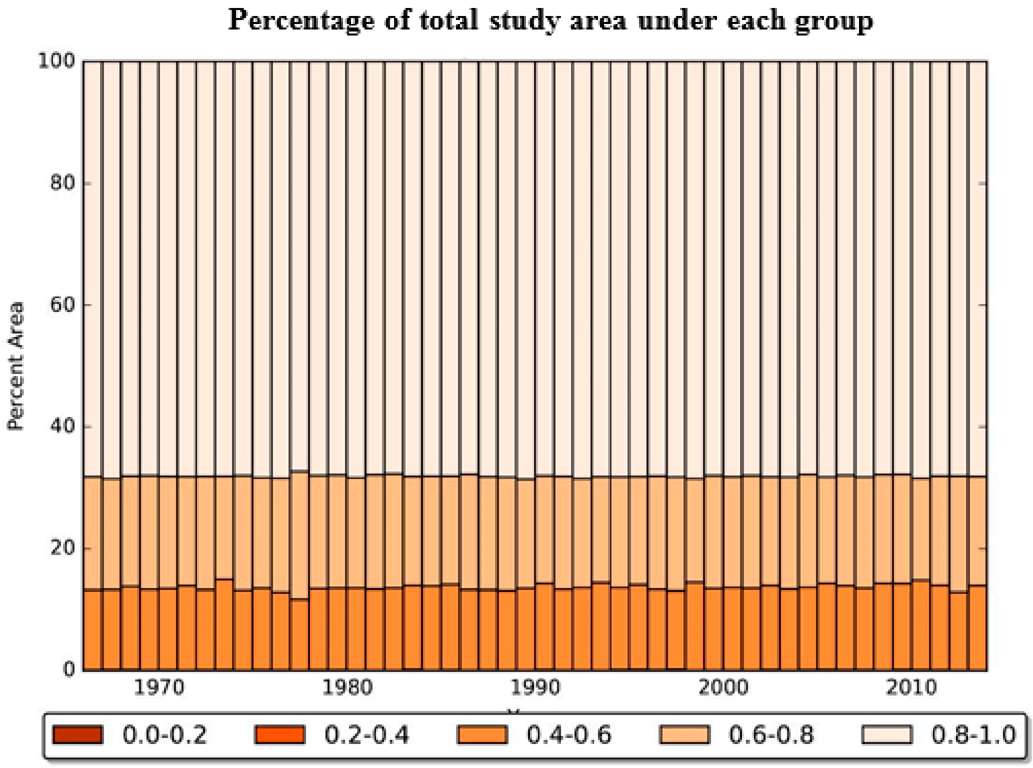

Figure S16: Percentage of total study area under different categories of watershed health (five categories defined in increments of 0.2, with respect to Nitrite+Nitrate) using the ensemble model during the period 1966-2014.

S.2.3 Watershed health for Orthophosphate

Orthophosphate (parameter code: 00671) values were available at a total of 49 stations over the study region. Following Hoque et al. (2012), observed samples were used to reconstruct a continuous daily time series of Orthophosphate loads. Using a standard of 0.1 mg/L (US EPA, 1986) the annual series of risk measures were obtained at each station. These annual series were used as target variables (y) during the training phase of machine learning models. The inputs (X) were attributes collected over areas draining to USGS-NAWQA stations. The machine learning

models were trained using 5-fold CV using data at 80% of the stations. Data at remaining 20% of the stations were used as test set.

Performances of ML models – Testing phase

Figure S17 shows the performance of three machine learning models, i.e., random forest regression (R^2=0.26, Figure S17a), gradient boosting regression (R^2=0.57, Figure S17b), and AdaBoost regression (R^2=0.32, Figure S17c), on the test dataset. Unlike in other cases, Bayesian regression resulted in poor model performance (R^2< -22, Figure S17d) and therefore was not included in the ensemble model. Results indicated that gradient boosting model provided relatively good fit, but for all three models there was significant scatter about the best fit line.

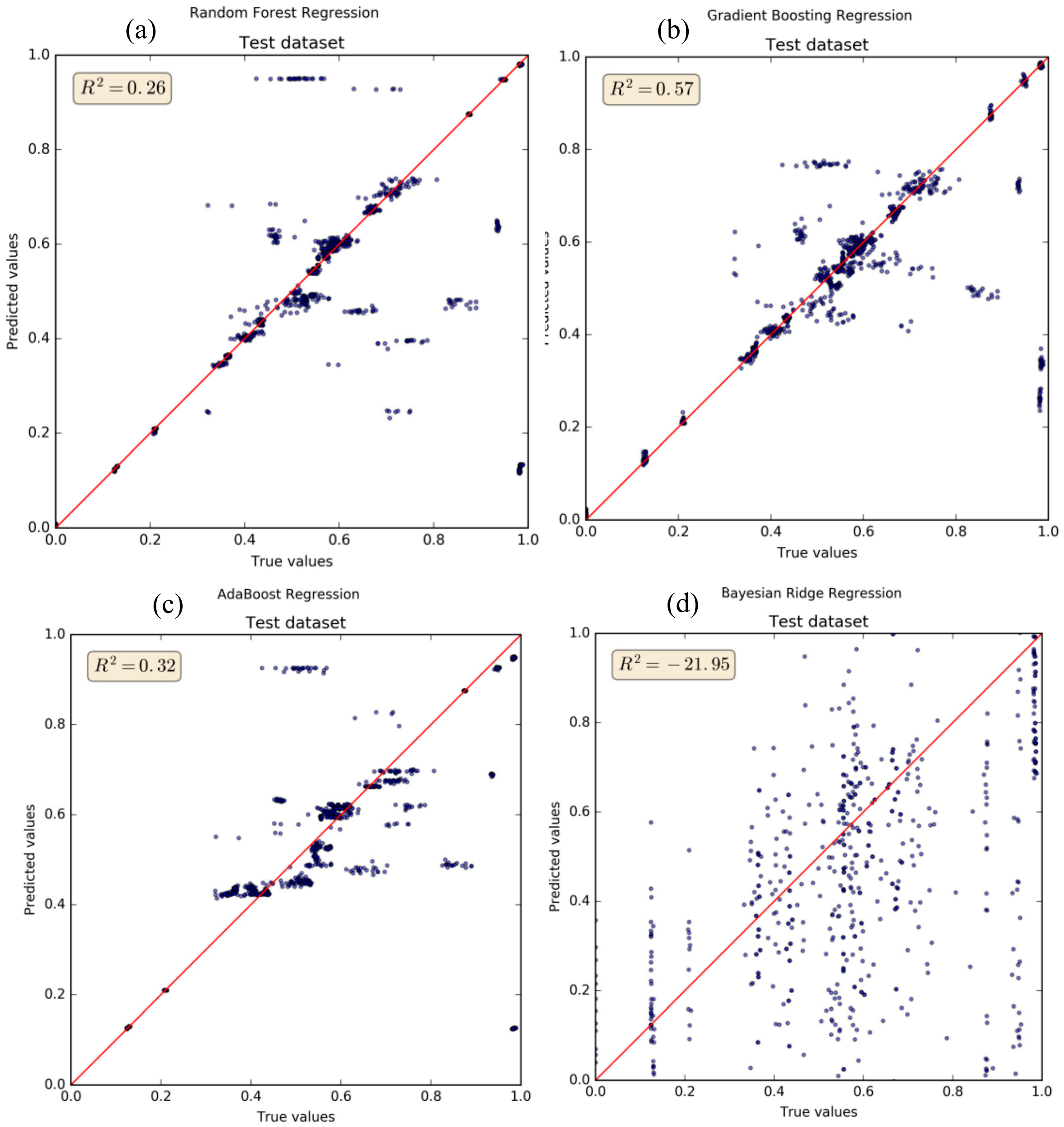

Figure S17: Scatter plot of reference WH versus predicted watershed health (with respect to Orthophosphate) using (a) random forest regression, (b) gradient boosting regression, (c) AdaBoost regression, and (d) Bayesian ridge regression at USGS-NAWQA stations that were used as test set. The values for each year during the study period (1966-2014) are shown.

Important explanatory variables – Random forest model

Figure S18 shows the top 15 out of 83 variables that were important in explaining watershed health (with respect to Orthophosphate) according to the trained random forest model. Results indicate that along with percentage of area under agricultural and forest land use, variables such as longitude, percentage area under hydrologic soil group B, and fertilizer sales were the most important.

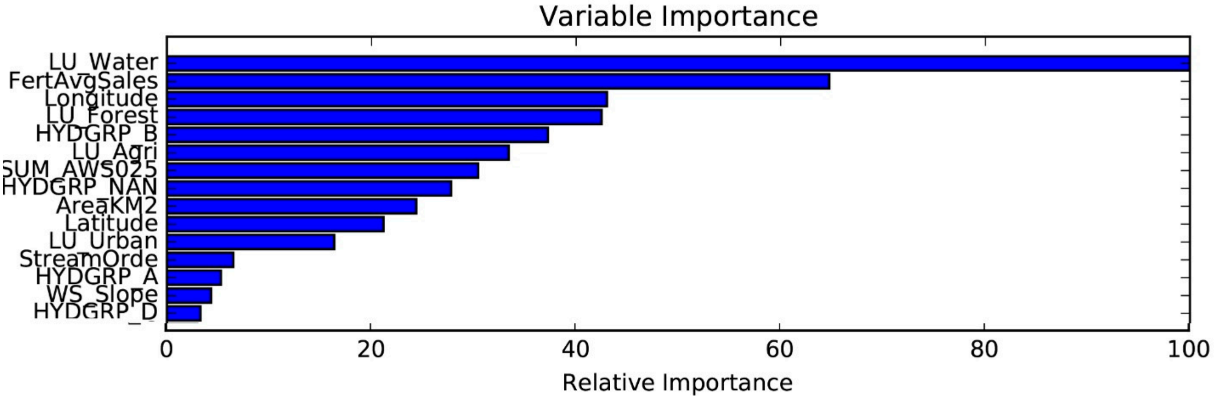

Figure S18: Variable importance for top 15 out of 83 explanatory variables according to random forest model trained on watershed health (for Orthophosphate) at USGS-NAWQA stations.

Ensemble model – Testing phase

To improve the overall predictive power, an ensemble approach was used. As in cases discussed above, a random forest model with 50 decision trees was trained using outputs of the three individual regression models at training stations as explanatory variables (X) and reference values (i.e., risk measures computed from observed data) at those stations as the target (y). The model was trained using 5-fold CV, and evaluated on the test-dataset. Figure S 19 shows the comparison

of reference values of watershed health (for orthophosphate) versus ensemble model predictions. The R^2 value was 0.99 for the ensemble model, and the scatter about the best fit line was considerably small when compared to individual models (see Figure S17).

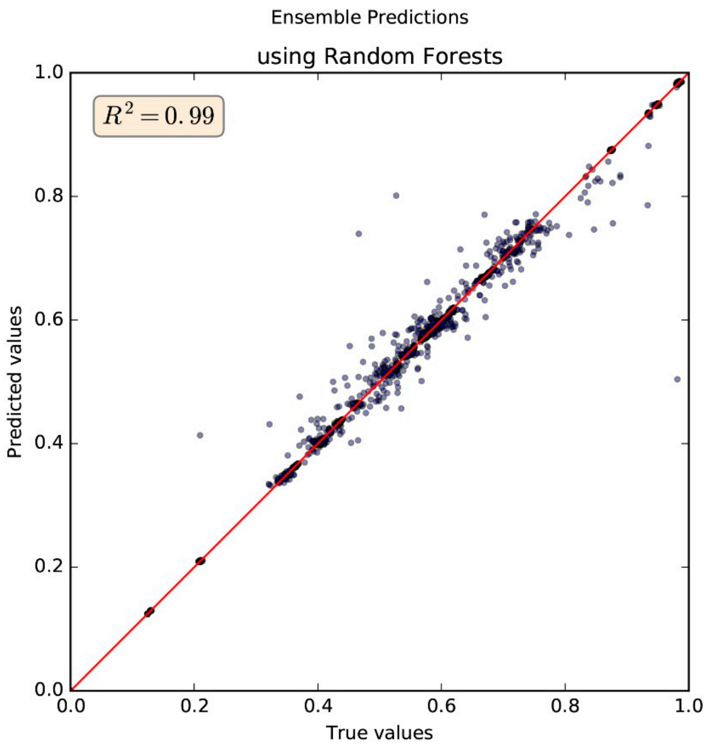

Figure S19: Scatter plot of reference versus ensemble model predictions of watershed health (with respect to Orthophosphate) at USGS-NAWQA stations that were used as test set. The values for each year during the study period (1966-2014) are shown.

<u>Variation of spatial extent of WH measure with time</u>

The variation of percentage of total area during the study period (1966-2014) under five discrete watershed health categories are shown in Figure S 20. Areas with moderate watershed health (0.4-0.6) were dominant with about 60% coverage. About 3% of the total study area had watershed

health in the range of 0.2 to 0.4, 60% of the area was in the range of 0.4 to 0.6, 29% of the area was in the range 0.6 to 0.8, and 8% in the range 0.8 to 1.0. The percentage area in the above three groups (0.2-0.4, 0.6-0.8, and 0.8 to 1.0) remained relatively unchanged over the study period. No portion of the study area had poor watershed health (0.0-0.2). There were small variations for the area under moderate watershed health (0.4-0.6), but did not have a long-term trend. The results in Figure S 20 correspond to the chosen standard of 0.1 mg/L for Orthophosphate.

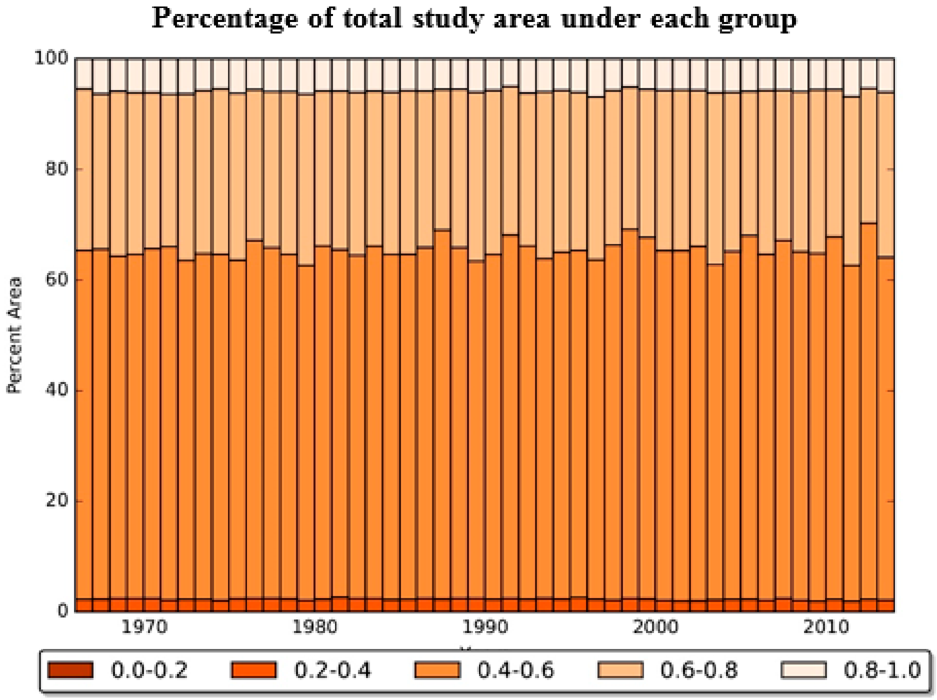

Figure S20: Percentage of total study area under different categories of watershed health (five categories defined in increments of 0.2, with respect to Orthophosphate) using the ensemble model during the period 1966-2014.
